# The unraveling of balanced complexes in metabolic networks

**DOI:** 10.1038/s41598-023-32666-6

**Published:** 2023-04-07

**Authors:** Damoun Langary, Anika Küken, Zoran Nikoloski

**Affiliations:** 1 Systems Biology and Mathematical Modeling, Max Planck Institute of Molecular Plant Physiology, Potsdam, Germany; 2 Institute of Biochemistry and Biology, University of Potsdam, Potsdam, Germany

**Keywords:** Biochemistry, Biotechnology, Chemical biology, Computational biology and bioinformatics

## Abstract

Balanced complexes in biochemical networks are at core of several theoretical and computational approaches that make statements about the properties of the steady states supported by the network. Recent computational approaches have employed balanced complexes to reduce metabolic networks, while ensuring preservation of particular steady-state properties; however, the underlying factors leading to the formation of balanced complexes have not been studied, yet. Here, we present a number of factorizations providing insights in mechanisms that lead to the origins of the corresponding balanced complexes. The proposed factorizations enable us to categorize balanced complexes into four distinct classes, each with specific origins and characteristics. They also provide the means to efficiently determine if a balanced complex in large-scale networks belongs to a particular class from the categorization. The results are obtained under very general conditions and irrespective of the network kinetics, rendering them broadly applicable across variety of network models. Application of the categorization shows that all classes of balanced complexes are present in large-scale metabolic models across all kingdoms of life, therefore paving the way to study their relevance with respect to different properties of steady states supported by these networks.

## Introduction

The last two decades have witnessed the generation of large-scale metabolic networks along with the development of computational approaches within the constraint-based modeling framework that facilitate insights in the genotype–phenotype map^1^. Metabolic networks include the entirety of biochemical reactions through which multiple species (e.g. metabolites, metabolite-enzyme complexes) are taken up from the environment and/or are transformed into the building blocks of biological systems. Since the first stoichiometric models of *E. coli*^2,3^, more refined metabolic models have been generated not only by increasing the gene and reaction coverage, but also by considering interactions of multiple cell types, tissues, organs in an organism^4^ as well as interactions between organisms in communities^5^.

The success of the constraint-based modeling framework can in part be ascribed to: (1) invoking the simplifying assumption of steady state for the concentration of species and (2) taking a flux-centered view that simplifies the metabolic constraints to a system of linear equations, in terms of the reaction rates (i.e. fluxes). The system of linear equation can be readily derived solely based on the stoichiometry of the analyzed network. Together with the capacity to include natural constraints on fluxes, these simplifications allow prediction of metabolic phenotypes with an assumed objective by employing classical convex optimization techniques (e.g. as done in flux balanced analysis^6^).

The same quest for linearity is followed in chemical reaction network theory (CRNT)^7^. Like constraint-based modeling, CRNT also invokes the steady-state constraints; however, CRNT takes a concentration-centered view and often makes the simplifying assumption that the modeled reactions follow the classical mass action kinetics^8^. As a result, one obtains a system of polynomial equations in terms of the species’ concentrations which can be analyzed at different levels of abstraction. For instance, linearity in CRNT is achieved due to the consideration of complexes, corresponding to the substrate/product side of each modelled reaction. This allows the rewriting of the systems of polynomial equation into linear equations in terms of the monomials, arising from mass action kinetics, parameterized by the rate constants of the assumed mass action kinetics.

**Figure 1:**
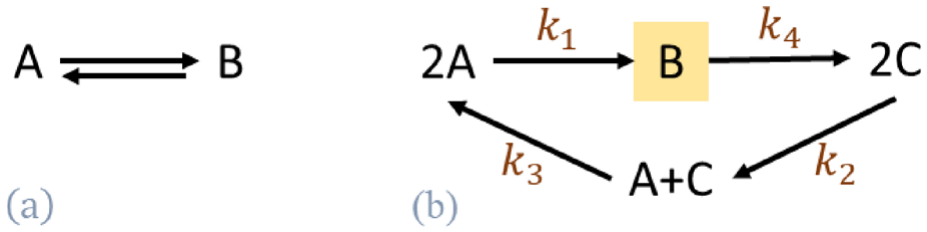
The distinction of three notions related to balanced complexes. (1) The diagram (**a**) depicts a complex balanced network. (2) The network in diagram (**b**) is not complex balanced. However, assuming mass action kinetics and under the particular condition ${k}_{1}{k}_{2}={{k}_{3}}^{2}$, all complexes are balanced at steady state. (3) The complex B [highlighted in (**b**)] is a balanced complex at all steady states regardless of the kinetic law governing the network.

As a consequence, CRNT has relied on linear algebraic tools to address very general questions about whether or not a dynamical property arises given any or some choices for the value of parameters^9,10^. For instance, one of the seminal results in CRNT deals with so-called complex balanced networks in which every complex is balanced, in the sense that the sums of reaction rates using the complex as a substrate or product are the same at every steady state supported by the network. Complex balanced networks have been shown not to exhibit exotic dynamic behavior, including capacity for multistationarity^9^ or presence of species showing robust concentrations^11^. Determining if a network is complex balanced can be done by calculating the deficiency of the network, relying solely on the structure of a given network^7,9^. We note that a network can be complex balanced irrespective of the imposed reaction kinetics. For instances, the network on Fig. 1a is of deficiency zero and hence complex balanced, for any choice of reaction kinetics.

However, balanced complexes are not only present in complex balanced networks. For instance, under the steady-state assumption, a complex including a species that does not occur in any other complex of a given network is balanced^12,13^. For instance, the network on Fig. 1b is not complex balanced, but has one balanced complexes for any choice of reaction kinetics—complex B. We note that the network on Fig. 1b endowed with mass action kinetics can be complex balanced for particular choice of reaction constants (see Section S3 of Supplementary Information).

Therefore, it is natural to ask the following three questions: (1) Can balanced complexes be efficiently computed in networks by using only the stoichiometric network representation of arbitrary kinetics? Affirmative answer to this question would imply that the so-identified complexes are balanced irrespective of the reaction kinetics. (2) What are the effects that realistic flux constraints have on presence of balanced complexes? Answering this question will specify if and to what extent balanced complexes may persist upon modifying flux bounds. (3) What are the network mechanisms that lead to the presence of balanced complexes with and without invoking flux constraints? Addressing this question is expected to provide new direction in reconciling the flux- and concentration-centered views, typical for the constraint-based and CRNT approaches. In addition, it will also help in shedding light on recent developments based on constraint-based linear programming approaches to identify balanced complexes in networks with arbitrary as well as mass action kinetics^12^. Since it has also been shown that balanced complexes can be used for effective reduction of large-scale metabolic models, answers to the abovementioned questions can help in addressing this problem that has gained some renewed interest^14^.

Here, we consider networks with arbitrary kinetics and provide a categorization of balanced complexes. The categorization delineates the contribution of network structure and flux constraints in categorizing a balanced complex into one of the four classes. We also formulate feasibility problems whose solutions can be used to categorize a balanced complex in a respective class. Our results also provide conditions that preclude the presence of particular class of balanced complexes irrespective of the kinetics that reactions may assume. In addition, by analyzing high-quality metabolic networks across kingdoms of life we show that all classes of balanced complexes are prevalent and, therefore, relevant for properties of the supported steady states.

## Material and methods

### Preliminaries on chemical reaction network theory (CRNT)

In the classical CRNT literature^9,10,15–18^, a chemical reaction network (CRN) is usually defined by the 3-tuple $G=\left(\mathcal{S},\mathcal{C},\mathcal{R}\right)$, where $\mathcal{S}$ is a set of $m$ *species/metabolites* and $\mathcal{C}$ is a set of $n$ *complexes*, whose elements $C\in \mathcal{C}$ can be regarded as multisets of species, $C\in {\mathbb{N}}^{\mathcal{S}}$. The set $\mathcal{R}$ is formally defined as a symmetric relation between network complexes, $\mathcal{R}\subset \mathcal{C}\times \mathcal{C}$; each element $\left({C}_{i},{C}_{j}\right)\in \mathcal{R}$ symbolizes the conversion of complex ${C}_{i}$ to ${C}_{j}$ or vice versa, and is referred to as a *reaction* of the network.

Throughout this manuscript, the standard basis for ${\mathbb{R}}^{n}$ will be denoted $\left\{{\mathbf{e}}_{1},{\mathbf{e}}_{2},\ldots ,{\mathbf{e}}_{n}\right\}$, where

$${\mathbf{e}}_{j}={\left[0\,\cdots\,0\,1\,0\,\cdots 0\right]}^{T} ,$$

is an $n$-vector with a unit value at the *j*th entry and zero entries elsewhere. Assuming some arbitrary ordering on the sets of species $({S}_{1},\ldots ,{S}_{m})$, complexes $\left({C}_{1},\ldots ,{C}_{n}\right)$, and reactions $({R}_{1},\ldots {R}_{r})$, any complex ${C}_{j}\in \mathcal{C}$ can be alternatively represented by a vector ${\mathbf{e}}_{j}\in {\mathbb{R}}^{n}$ indexing its position in the ordered set, and at the same time associated with a unique vector ${\mathbf{y}}_{j}\in {\mathbb{R}}^{m}$, representing its species content. This defines the following mapping

1 $${\mathbf{e}}_{j} \mapsto {\mathbf{y}}_{j} \in {\mathbb{R}}^{m} { },\;\;1 \le j \le n{ }.$$

As a result, any given CRN is associated with a matrix defined as a compilation of vectors $\mathbf{Y}=\left[{\mathbf{y}}_{1} \cdots {\mathbf{y}}_{n}\right]$, referred to as the *stoichiometric map*. In a similar fashion, one can associate any reaction ${R}_{k} : {C}_{{k}_{1}}\to {C}_{{k}_{2}}\in \mathcal{R}$ with vectors ${\mathbf{a}}_{k}={\mathbf{e}}_{{k}_{2}}-{\mathbf{e}}_{{k}_{1}}\in {\mathbb{R}}^{n}$ and ${\mathbf{n}}_{k}={\mathbf{y}}_{{k}_{2}}-{\mathbf{y}}_{{k}_{1}}\in {\mathbb{R}}^{m}$. Assembling these vectors in the same order gives the *incidence matrix* $\mathbf{A}=\left[{\mathbf{a}}_{1} \cdots {\mathbf{a}}_{r}\right]$ and the *stoichiometry matrix* $\mathbf{N}=\left[{\mathbf{n}}_{1} \cdots {\mathbf{n}}_{r}\right]$, respectively. If follows that $\mathbf{N}=\mathbf{Y}\mathbf{A} .$ Since we consider the reactions as potentially reversible entities, we keep to the convention that no two distinct columns in $\mathbf{A}$ (or in $\mathbf{N}$) are collinear. The reader shall not confuse this with the classical convention in CRNT, where reversible reactions are presented by two separate reaction vectors.

The dynamics of the CRN is formulated by the following equation

2 $$\frac{d}{dt}{\mathbf{x}} = {\mathbf{N}} {\mathbf{v}} = {\mathbf{Y}} {\mathbf{A}} {\mathbf{v}} ,$$

where $\mathbf{x}\in {\mathbb{R}}_{+}^{m}$ is the vector of species concentrations and $\mathbf{v}=\mathbf{v}\left(\mathbf{x}\right)\in {\mathbb{R}}^{r}$ is the vector of kinetic rates, referred to as *fluxes*, which is generally a nonlinear function of $\mathbf{x}$. A positive flux ${v}_{k}$ for reaction ${R}_{k} : {C}_{{k}_{1}}\to {C}_{{k}_{2}}\in \mathcal{R}$ signifies the net conversion of ${C}_{{k}_{1}}$ to ${C}_{{k}_{2}}$, while a negative flux ${v}_{k}$ marks the net conversion of ${C}_{{k}_{2}}$ to ${C}_{{k}_{1}}$. The *flux distribution* $\mathbf{v}$ is often bounded by an upper bound ${\mathbf{v}}_{\text{u}}$ and a lower bound ${\mathbf{v}}_{\text{l}}$ as follows

3 $${\mathbf{v}}_{{\text{l}}} \le {\mathbf{v}} \le {\mathbf{v}}_{{\text{u}}} .$$

By convention, ${\mathbf{v}}_{\text{u}}$ has strictly positive entries for all reactions. The set of *irreversible reactions* ${\mathcal{R}}^{\text{irr}}\subseteq \mathcal{R}$ can be determined by the nonnegative elements of ${\mathbf{v}}_{\text{l}}$, that is

$${\mathcal{R}}^{{{\text{irr}}}} = \left\{ {R \in {\mathcal{R}} | v_{l,R} \ge 0} \right\} ,$$

where ${v}_{{\text{l}},R}$ is the designated lower bound on flux through reaction $R$.

We say the system is operating in a *canonical flux regime*, if ${v}_{{\text{l}},R}=0, \forall R\in {\mathcal{R}}^{\text{irr}}$, that is, no irreversible reaction is forced to take a strictly positive flux value. The set of *reversible reactions* is simply defined as the complement of ${\mathcal{R}}^{\text{irr}}$ in $\mathcal{R}$, i.e. ${\mathcal{R}}^{\text{rev}}=\mathcal{R}\setminus {\mathcal{R}}^{\text{irr}}$. The system is said to be operating in an unbounded flux regime, if ${v}_{{\text{u}},R}=\infty,\, \forall R\in \mathcal{R};\,{v}_{{\text{l}},R}=-\infty,\, \forall R\in {\mathcal{R}}^{\text{rev}}$. One can always choose an ordering of the reactions in which elements of ${\mathcal{R}}^{\text{rev}}$ precede those of ${\mathcal{R}}^{\text{irr}}$. In this case, the incidence matrix $\mathbf{A}$ can be partitioned into matrix blocks $\mathbf{A}=\left[{\mathbf{A}}^{\text{rev}}{\mathbf{A}}^{\text{irr}}\right]$.

For the system to be at steady state, the flux vector $\mathbf{v}$ must lie in the nullspace of matrix $\mathbf{N}$, that is

4 $${\mathbf{N}} {\mathbf{v}} = {\mathbf{Y}} {\mathbf{A}} {\mathbf{v}} = 0 .$$

The intercept of the inequality constraints in Eq. (3) and equality constraint in Eq. (4) defines a convex set of feasible vectors $\mathbf{v}$, *the steady state flux set*, denoted $\mathcal{F}\left(G\right)=\left\{\mathbf{v}\in {\mathbb{R}}^{r} | \mathbf{Y}\mathbf{A}\mathbf{v}=0,\, {\mathbf{v}}_{\text{l}}\le \mathbf{v}\le {\mathbf{v}}_{\text{u}}\right\}$. In a canonical and unbounded flux regime, the feasible set forms a polyhedral cone, referred to as the *steady state flux cone*.

For any $R\in \mathcal{R}$, we say $R$ is a *blocked reaction* in $G$, if for all the feasible set distributions $\mathbf{v}\in \mathcal{F}\left(G\right)$, there is zero flux through $R$, that is, ${v}_{R}=0$. This is a commonly occurring phenomenon in metabolic networks, especially in scenarios when flux bounds and/or optimization of particular objectives are imposed. Similarly, we say a reaction $R\in \mathcal{R}$ is *fixated* at some flux value $f$, if for all the flux distributions $\mathbf{v}\in \mathcal{F}\left(G\right)$, the flux through $R$ is unchanged, namely, ${v}_{R}=f, \forall \mathbf{v}\in \mathcal{F}\left(G\right)$. Clearly, any blocked reaction is one fixated at zero.

### Balanced complexes

An important class of steady state fluxes are the so-called *complex-balanced steady states*, which were introduced by Horn and Jackson^16,19^ as a generalization of detailed balanced steady states. At a complex-balanced steady state, the flux distribution satisfies

5 $${\mathbf{A}} {\mathbf{v}} = 0.$$

Equation (5) constrains $\mathbf{v}$ to lie in $\mathrm{ker}\mathbf{A}$, which is generally a subset of $\mathrm{ker}\mathbf{N}$, and, hence, is a stronger condition compared to Eq. (4). From a conceptual viewpoint, complex-balanced steady states are those in which the algebraic sum of the total flux entering and leaving each complex equals zero. In more technical terms, the flux vector is composed of a number of cyclic generators^20^, i.e. nonstoichiometric elementary flux modes^21^. Of particular interest are systems for which all steady states are complex-balanced, that is, Eq. (4) can be seamlessly replaced by Eq. (5). This class of systems-referred to as *complex-balanced systems*- are well-studied in the classical body of work on CRNT, and very strong results have been derived for them, most notably the well-known Deficiency Zero Theorem^9^.

Unfortunately, in most cases those strong results cannot be applied to the majority of real-world metabolic networks: Due to the more complex nature of such systems, often not all but only some complexes are balanced at steady state. However, recent studies have shown that, even in absence of full complex-balancing, existence of balanced complexes can be exploited to obtain significant network reductions and throw some light on the steady states^12,13,22^. This gives rise to the question regarding the underlying mechanisms that lead to the formation of complex balancing in metabolic networks.

Let us next give a formal definition of a balanced complex. For a given matrix $\mathbf{X}$, let ${\mathbf{X}}^{:i}$ and ${\mathbf{X}}^{j:}$ denote the ${i}$th column and ${j}$th row of $\mathbf{X}$, respectively. A complex ${C}_{j}\in \mathcal{C}$ is defined to be a *balanced complex* (BC) for network $G$, if ${\mathbf{A}}^{j:}\mathbf{v}\equiv 0$ for all $\mathbf{v}\in \mathcal{F}\left(G\right)$. Given how incidence matrix $\mathbf{A}$ is constructed, this definition complies with the notion that the algebraic sum of fluxes entering and leaving ${C}_{j}$ must be always zero at any steady state.

### Primal–dual formulations

To investigate whether a complex ${C}_{j}\in \mathcal{C}$ is a BC, one may form and solve the following two linear programs (LP)

6 $$\begin{array}{*{20}l} {\begin{array}{*{20}l} {\mathop {{\text{minimize}}}\limits_{{{\mathbf{v}} \in {\mathbb{R}}^{r} }} { }} \hfill & { {\mathbf{e}}_{j}^{{\text{T}}} {\mathbf{A}}\user2{ }{\mathbf{v}}} \hfill \\ {{\text{subject}}\;{\text{to }}} \hfill & { \begin{array}{*{20}l} {{\mathbf{Y}} {\mathbf{A}} {\mathbf{v}} = 0} \hfill \\ {{\mathbf{v}}_{{\text{l}}} \le {\mathbf{v}} \le {\mathbf{v}}_{{\text{u}}} } \hfill \\ \end{array} } \hfill \\ \end{array} } \hfill \\ \end{array},$$

7 $$\begin{array}{*{20}l} {\mathop {{\text{maximize}}}\limits_{{{\mathbf{v}} \in {\mathbb{R}}^{r} }} { }} \hfill & {{\mathbf{e}}_{j}^{{\text{T}}} {\mathbf{A}}\user2{ }{\mathbf{v}}} \hfill \\ {{\text{subject}}\;{\text{to }}} \hfill & { \begin{array}{*{20}l} {{\mathbf{Y}} {\mathbf{A}} {\mathbf{v}} = 0} \hfill \\ {{\mathbf{v}}_{{\text{l}}} \le {\mathbf{v}} \le {\mathbf{v}}_{{\text{u}}} } \hfill \\ \end{array} } \hfill \\ \end{array} .$$

The definition implies that ${C}_{j}\in \mathcal{C}$ is a BC if and only if both above LPs have zero optimal values. The optimization problems (6) and (7) have a Lagrange dual of the following form [derivation in Section S1.2 of the Supplementary Information]

8 $$\begin{array}{*{20}l} {\mathop {{\text{maximize}}}\limits_{{{{\varvec{\uplambda}}}_{{\text{u}}} \user2{ }, {{\varvec{\uplambda}}}_{{\text{l}}} \in {\mathbb{R}}^{r} , {{\varvec{\upzeta}}} \in {\mathbb{R}}^{m} }} \;} \hfill & {{{\varvec{\uplambda}}}_{{\text{l}}}^{{\text{T}}} {\mathbf{v}}_{{\text{l}}} - {{\varvec{\uplambda}}}_{{\text{u}}}^{{\text{T}}} {\mathbf{v}}_{{\text{u}}} } \hfill \\ {{\text{subject}}\;{\text{to}}\; } \hfill & {\begin{array}{*{20}l} {{\mathbf{A}}^{{\text{T}}} {\mathbf{e}}_{j} - {\mathbf{A}}^{{\text{T}}} {\mathbf{Y}}^{{\text{T}}} {{\varvec{\upzeta}}} + {{\varvec{\uplambda}}}_{{\text{u}}} - {{\varvec{\uplambda}}}_{{\text{l}}} = 0} \hfill \\ {{{\varvec{\uplambda}}}_{{\text{u}}} \user2{ }, {{\varvec{\uplambda}}}_{{\text{l}}} \ge 0 } \hfill \\ \end{array} } \hfill \\ \end{array}. $$

9 $$\begin{array}{*{20}c} {\begin{array}{*{20}l} {\mathop {{\text{maximize}}}\limits_{{{{\varvec{\uplambda}}}_{{\text{u}}} \user2{ }, {{\varvec{\uplambda}}}_{{\text{l}}} \in {\mathbb{R}}^{r} , {{\varvec{\upzeta}}} \in {\mathbb{R}}^{m} }} {{\varvec{\uplambda}}}_{{\text{l}}}^{{\text{T}}} {\mathbf{v}}_{{\text{l}}} - {{\varvec{\uplambda}}}_{{\text{u}}}^{{\text{T}}} {\mathbf{v}}_{{\text{u}}} } \hfill \\ {{\text{ subject to }} \begin{array}{*{20}l} { - {\mathbf{A}}^{{\text{T}}} {\mathbf{e}}_{j} + {\mathbf{A}}^{{\text{T}}} {\mathbf{Y}}^{{\text{T}}} {{\varvec{\upzeta}}} + {{\varvec{\uplambda}}}_{{\text{u}}} - {{\varvec{\uplambda}}}_{{\text{l}}} = 0} \hfill \\ {{{\varvec{\uplambda}}}_{{\text{u}}} \user2{ }, {{\varvec{\uplambda}}}_{{\text{l}}} \ge 0 } \hfill \\ \end{array} } \hfill \\ \end{array} } \\ \end{array}.$$

Clearly, given the linear constraints, strong duality holds and the duality gap is zero^23^; therefore, a complex ${C}_{j}\in \mathcal{C}$ is a BC if and only if both Problems (8) and (9) have optimal values equal to zero.

## Results and discussion

### Generalization of trivial BCs

Taking into consideration the stoichiometry of a chemical reaction network, it is straightforward to see how some balanced complexes may emerge. A complex ${C}_{j}\in \mathcal{C}$ is *trivially-balanced* (a *trivial BC*), if there exist some species ${S}_{i}\in \mathcal{S}$ that appears only in ${C}_{j}$ and nowhere else in the network^12^. Given the structure of the stoichiometric map $\mathbf{Y}$, it follows that ${\mathbf{Y}}^{i:}={{\mathbf{e}}_{j}}^{\mathrm{T}}$. Hence, the steady state equation $\mathbf{Y}\mathbf{A}\mathbf{v}=0$ immediately yields ${{\mathbf{e}}_{j}}^{\mathrm{T}}\mathbf{A}\mathbf{v}\equiv 0 , \forall \mathbf{v}\in \mathcal{F}\left(G\right)$.

One can readily generalize the idea behind the formation of a trivial BC to potentially arrive at a larger class of balanced complexes in the network. Intuitively, the vector ${\mathbf{e}}_{j}$ does not have to appear explicitly as a row in $\mathbf{Y}$; all it takes is for ${\mathbf{e}}_{j}$ to be a linear combination of rows in $\mathbf{Y}$, that is

10 $$\exists {{\varvec{\upzeta}}} \in {\mathbb{R}}^{m} : {\mathbf{e}}_{j} = {\mathbf{Y}}^{{\text{T}}} {{\varvec{\upzeta}}};$$

it then follows that ${{\mathbf{e}}_{j}}^{\mathrm{T}}\mathbf{A}\mathbf{v}={{\varvec{\upzeta}}}^{\mathrm{T}}\mathbf{Y}\mathbf{A}\mathbf{v}=0,\, \forall \mathbf{v}\in \mathcal{F}\left(G\right) .$ We remark that Eq. (10) can potentially explain a higher number of balanced complexes in the network, and it fully contains all the trivial BCs.

**Figure 2:**
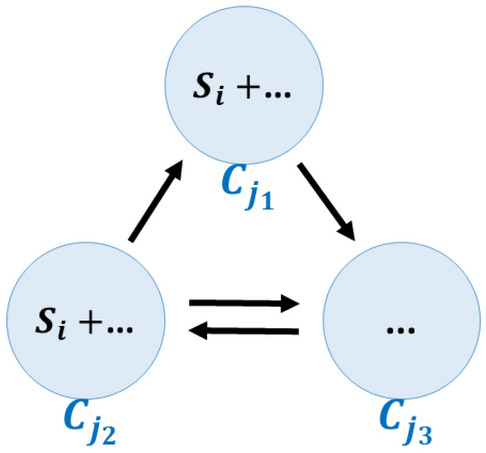
The topology contributing to the formation of a BC. A small linkage class of a network comprising three complexes is shown. The species ${S}_{i}$ is assumed to be present in complexes ${C}_{{j}_{1}}$ and ${C}_{{j}_{2}}$, but nowhere else in the network.

One may take another step and further generalize this notion to potentially identify an even larger class of BCs. Note that all BCs predicted by Eq. (10) have purely stoichiometric grounds, derived from stoichiometric map $\mathbf{Y}$. However, the topological structure of a network, pinpointed by matrix $\mathbf{A}$, can also play a role in the formation of BCs. Let us next consider a simple example, in which some species ${S}_{i}\in \mathcal{S}$ makes a unimolecular appearance in complexes ${C}_{{j}_{1}}$ and ${C}_{{j}_{2}},$ but is absent elsewhere in the network. Assume additionally that ${C}_{{j}_{1}}, {C}_{{j}_{2}}$ form a linkage class with another complex, namely ${C}_{{j}_{3}}$. The situation is shown in Fig. 2.

On the one hand, ${\mathbf{Y}}^{i:}={\left({\mathbf{e}}_{{j}_{1}}+{\mathbf{e}}_{{j}_{2}}\right)}^{\mathrm{T}}$ and the steady state condition for species ${S}_{i}$ imply that $\left({\mathbf{A}}^{{j}_{1}:}+{\mathbf{A}}^{{j}_{2}:}\right)\mathbf{v}=0,\, \forall \mathbf{v}\in \mathcal{F}\left(G\right)$; on the other hand, the closedness of the linkage class requires that ${\mathbf{A}}^{{j}_{1}:}+{\mathbf{A}}^{{j}_{2}:}+{\mathbf{A}}^{{j}_{3}:}={0}^{\mathrm{T}}$. Thus, it follows that ${\mathbf{A}}^{{j}_{3}:}\mathbf{v}=0,\, \forall \mathbf{v}\in \mathcal{F}\left(G\right)$, that is,${C}_{{j}_{3}}$ is a BC. Note that the balancing of ${C}_{{j}_{3}}$ has nothing to do with the species in it, but with its connection to other complexes.

Formalizing this generalization relies on integrating the linkage structure of the network, which is closely interlinked with the left nullspace of the incidence matrix $\mathbf{A}$, into Eq. (10). Let $G$ be a closed network composed of ${\ell }$ linkage classes ${L}_{1},\ldots ,{L}_{\ell }$. The left nullspace of $\mathbf{A}$, i.e. $\mathrm{ker}{\mathbf{A}}^{\mathrm{T}}$ has a basis of the form $\left\{{\mathbf{u}}_{1},\ldots ,{\mathbf{u}}_{\ell }\right\}$, where ${\mathbf{u}}_{l}={\sum }_{\iota : {C}_{\iota }\in {L}_{l}} {\mathbf{e}}_{\iota } ,\,1\le\,l\le\,{\ell }$. Next, define the matrix $\mathbf{U}\in {\mathbb{R}}^{n\times {\ell }}$ as $\mathbf{U}=\left[{\mathbf{u}}_{1}\cdots {\mathbf{u}}_{\ell }\right]$. Now, let a complex ${\mathbf{e}}_{j}$ have the following form

11 $$\exists {{\varvec{\upzeta}}} \in {\mathbb{R}}^{m} ,\user2{ }{{\varvec{\upxi}}} \in {\mathbb{R}}^{\ell } : {\mathbf{e}}_{j} = {\mathbf{Y}}^{{\text{T}}} {{\varvec{\upzeta}}} + {\mathbf{U}} {{\varvec{\upxi}}};$$

it follows that ${{\mathbf{e}}_{j}}^{\mathrm{T}}\mathbf{A}\mathbf{v}={{\varvec{\upzeta}}}^{\mathrm{T}}\mathbf{Y}\mathbf{A}\mathbf{v}+{{\varvec{\upxi}}}^{\mathrm{T}}\mathbf{U}\mathbf{A}\mathbf{v}=0 , \forall \mathbf{v}\in \mathcal{F}\left(G\right) .$ Equation (11) shall be viewed as a generalization of Eq. (10), which can potentially account for a higher number of BCs in the network.

### Factorizations of balanced complexes

Equation (11), like Eq. (10), provides a sufficient condition: If there exist vectors ${\varvec{\upzeta}}\in {\mathbb{R}}^{m},\,\boldsymbol{ }{\varvec{\upxi}}\in {\mathbb{R}}^{\ell }$ such that Eq. (11) holds for some ${\mathbf{e}}_{j}$, then complex ${C}_{j}\in \mathcal{C}$ is a BC. This poses the following question: Does Eq. (11) account for all BCs in the network? We seek to determine whether all BCs are created as a result of an interplay of stoichiometry and the linkage structure or there exist other factors contributing to the formation of BCs. We are also interested in establishing the conditions under which Eq. (11) would become a necessary and sufficient condition for BCs.

The dual formulation in Eqs. (8) and (9) helps address these questions. Before putting forward the result for the most general case, let us first present the following result, which introduces an intermediary step that elucidates the connection between these formulations.

#### Theorem 1

Suppose the network is operating under a canonical flux regime. There exist variables ${{\varvec{\upzeta}}}_{1},\boldsymbol{ }{{\varvec{\upzeta}}}_{2}\in {\mathbb{R}}^{m} ,\,\boldsymbol{ }\boldsymbol{ }{{\varvec{\upxi}}}_{1},\,\boldsymbol{ }{{\varvec{\upxi}}}_{2}\in {\mathbb{R}}^{{\ell }},\, {{\varvec{\uptheta}}}_{1},\,\boldsymbol{ }{{\varvec{\uptheta}}}_{2}\in {\mathbb{R}}^{n}$, such that

12 $$\left\{ { \begin{array}{*{20}l} {{\mathbf{e}}_{j} = {\mathbf{Y}}^{{\text{T}}} {{\varvec{\upzeta}}}_{1} + {\mathbf{U}} {{\varvec{\upxi}}}_{1} + {{\varvec{\uptheta}}}_{1} } \hfill \\ {{\mathbf{e}}_{j} = {\mathbf{Y}}^{{\text{T}}} {{\varvec{\upzeta}}}_{2} + {\mathbf{U}} {{\varvec{\upxi}}}_{2} - {{\varvec{\uptheta}}}_{2} } \hfill \\ {{\mathbf{A}}^{{{\text{revT}}}} {{\varvec{\uptheta}}}_{1} = 0} \hfill \\ {{\mathbf{A}}^{{{\text{irrT}}}} {{\varvec{\uptheta}}}_{1} \ge 0} \hfill \\ {{\mathbf{A}}^{{{\text{revT}}}} {{\varvec{\uptheta}}}_{2} = 0} \hfill \\ {{\mathbf{A}}^{{{\text{irrT}}}} {{\varvec{\uptheta}}}_{2} \ge 0} \hfill \\ \end{array} , } \right.$$

if and only if the complex ${C}_{j}\in \mathcal{C}$ is a BC. By convention, ${\mathbf{A}}^{\mathrm{irr}}$ denotes the columns of $\mathbf{A}$ corresponding to zero lower bounds in ${\mathbf{v}}_{\text{l}}$, and ${\mathbf{A}}^{\mathrm{rev}}$ is the complementary block in $\mathbf{A}$.

It is straightforward to see how Eq. (12) is a generalization of Eq. (11). In particular, any feasible solution of Eq. (11) for parameter values ${\varvec{\upzeta}},{\varvec{\upxi}}$ corresponds to a solution of Eq. (12) with feasible parameter values ${{\varvec{\upzeta}}}_{1}={{\varvec{\upzeta}}}_{2}={\varvec{\upzeta}},\, {{\varvec{\upxi}}}_{1}={{\varvec{\upxi}}}_{2}={\varvec{\upxi}},\, {{\varvec{\uptheta}}}_{1}={{\varvec{\uptheta}}}_{2}=0$. Note that irreversibility patterns play a key role in distinguishing the solution sets of Eqs. (12) and (11).

The following result holds for the most general case, i.e., also for cases, where the network is not operating under a canonical flux regime.

#### Theorem 2

A complex ${C}_{j}\in \mathcal{C}$ is a BC, if and only if there exist variables ${{\varvec{\upzeta}}}_{1},\,\boldsymbol{ }{{\varvec{\upzeta}}}_{2}\in {\mathbb{R}}^{m} ,\,\boldsymbol{ }\boldsymbol{ }{{\varvec{\uplambda}}}_{{\text{u}}1},\, {{\varvec{\uplambda}}}_{{\text{l}}1},\,\boldsymbol{ }{{\varvec{\uplambda}}}_{{\text{u}}2},\, {{\varvec{\uplambda}}}_{{\text{l}}2}\in {\mathbb{R}}^{r}$, such that

13 $$\left\{ { \begin{array}{*{20}l} {{\mathbf{A}}^{{\text{T}}} {\mathbf{e}}_{j} = {\mathbf{A}}^{{\text{T}}} {\mathbf{Y}}^{{\text{T}}} {{\varvec{\upzeta}}}_{1} + {{\varvec{\uplambda}}}_{{{\text{l}}1}} - {{\varvec{\uplambda}}}_{{{\text{u}}1}} } \hfill \\ {{\mathbf{v}}_{l}^{{\text{T}}} {{\varvec{\uplambda}}}_{{{\text{l}}1}} - {\mathbf{v}}_{u}^{{\text{T}}} {{\varvec{\uplambda}}}_{{{\text{u}}1}} = 0} \hfill \\ {{\mathbf{A}}^{{\text{T}}} {\mathbf{e}}_{j} = {\mathbf{A}}^{{\text{T}}} {\mathbf{Y}}^{{\text{T}}} {{\varvec{\upzeta}}}_{2} + {{\varvec{\uplambda}}}_{{{\text{u}}2}} - {{\varvec{\uplambda}}}_{{{\text{l}}2}} } \hfill \\ {{\mathbf{v}}_{l}^{{\text{T}}} {{\varvec{\uplambda}}}_{{{\text{l}}2}} - {\mathbf{v}}_{u}^{{\text{T}}} {{\varvec{\uplambda}}}_{{{\text{u}}2}} = 0} \hfill \\ {{{\varvec{\uplambda}}}_{{{\text{u}}1}} , {{\varvec{\uplambda}}}_{{{\text{l}}1}} ,\user2{ }{{\varvec{\uplambda}}}_{{{\text{u}}2}} , {{\varvec{\uplambda}}}_{{{\text{l}}2}} \ge 0{ }} \hfill \\ \end{array} . } \right.$$

Equation (13) brings into play the potential role of upper- and lower bounds on reaction fluxes as a final factor in the formation of balanced complexes.

It is not difficult to show how Eq. (13) can be viewed as a generalization of Eq. (12), and hence Eqs. (10) and (11). One only needs to define ${{\varvec{\uplambda}}}_{{\text{l}}t}={\mathbf{A}}^{\mathrm{T}}{{\varvec{\uptheta}}}_{t} , {{\varvec{\uplambda}}}_{{\text{u}}t}=0 ; t=\mathrm{1,2}$ to obtain an implicit form of Eq. (12). [refer to Section S1.4 of the Supplementary Information for mathematical details]. This shall not be surprising, as these relations are directly obtained from dual formulations in Eqs. (8) and (9).

### Categorization of BCs

In Eqs. (10), (11), (12) and (13), we presented four different formulations that can explain the emergence of a BC potentially as a combined effect of distinct factors, namely stoichiometry, linkage structure, irreversibility patterns and flux bounds, respectively. Each “*factorization*” can be viewed as a feasibility problem; let us next define the *solution set* of each factorization as the set of all complexes ${C}_{j}\in \mathcal{C}$, where vector ${\mathbf{e}}_{j}$ satisfies the feasibility problem for some parameter values. It is worth noting that the solution set of each factorization is a subset of the next one, in the abovementioned order of factorizations. Moreover, the solution set of Eq. (12) includes every existing BC in the network. This property paves the way to classify each BC of the network into one of the following four categories:

1. A complex ${C}_{j}\in \mathcal{C}$ is called a *strictly stoichiometric* BC, if it has a factorization of the form Eq. (10), i.e., $\exists {\varvec{\upzeta}}\in {\mathbb{R}}^{m} : {\mathbf{e}}_{j}={\mathbf{Y}}^{\mathrm{T}}{\varvec{\upzeta}} .$ This relation is referred to as a *strictly stoichiometric factorization* for the BC represented by vector ${\mathbf{e}}_{j}$.
2. A complex ${C}_{j}\in \mathcal{C}$ is called a *stoichiometric* BC, if it has a factorization of the form Eq. (11), i.e., $\exists {\varvec{\upzeta}}\in {\mathbb{R}}^{m},\,\boldsymbol{ }{\varvec{\upxi}}\in {\mathbb{R}}^{{\ell }} : {\mathbf{e}}_{j}={\mathbf{Y}}^{\mathrm{T}}{\varvec{\upzeta}}+\mathbf{U}{\varvec{\upxi}} .$ This relation is referred to as a *stoichiometric factorization* for the BC represented by vector ${\mathbf{e}}_{j}$.
3. A complex ${C}_{j}\in \mathcal{C}$ is called a *type-I nonstoichiometric* BC, if it has a factorization of the form Eq. (12), but no stoichiometric factorization. Equation (12) is called a *type-I nonstoichiometric factorization* for the BC represented by vector ${\mathbf{e}}_{j}$.
4. A complex $C\in \mathcal{C}$ is called a *type-II nonstoichiometric* BC, if it has a factorization of the form Eq. (13), but no factorization of the form Eq. (12). Equation (13) is called a *type-II nonstoichiometric factorization* for the BC represented by vector ${\mathbf{e}}_{j}$.

It follows directly from these definitions that any strictly stoichiometric BC/factorization is also a stoichiometric BC/factorization. Moreover, a balanced complex is labeled with the general term *nonstoichiometric BC*, if it has no stoichiometric factorization, regardless of whether it has an explicit factorization of the form Eq. (12) or an implicit factorization of the form Eq. (13).

### Characteristics of distinct BC categories

Recent studies show that balanced complexes can play an important role in the reduction of metabolic networks and simplification of steady state analysis^12,13^. The categorization of BCs into distinct classes not only helps identify the origin of the balancing in each case, but also enables one to study their properties. On the one hand, this equips us with an insight into how certain modification of a network may affect existing BCs. On the other hand, it may demonstrate if and how modeling inaccuracies may lead to a detection failure for certain existing BCs.

The strictly stoichiometric BCs constitute the most trivial class, whose formation relies merely on the network stoichiometry. This simplicity gives rise to an intrinsic form of robustness: As long as network complexes are unaltered, the balancing property of strictly stoichiometric BCs remains preserved, regardless of any potential changes in the network, e.g. even if the reactions in these networks are altered (referred to as different enzymatic regimes). In this case, the formation of strictly stoichiometric BCs roots solely in the steady state property for the species.

For the more general class of stoichiometric BCs, their formation not only relies on the network stoichiometry, but also partly on a topological feature, namely the linkage structure. They are affected neither by the irreversibility patterns in the network nor by the constraints on reaction rates. However, they may be altered under different enzymatic/catalytic regimes. Their balancing property is rooted not only in the steady state property but also in the conversion patterns (i.e. reaction structure) of the network.

The formation of nonstoichiometric BCs is a more sophisticated and at the same time more interesting phenomenon. They often emerge as a combined effect of several network features, including the stoichiometry and the linkage structure, but also irreversibility patterns as well as upper- and lower bounds on fluxes. In other words, these BCs carry information in the form of an equality constraint on reaction fluxes, which is not encoded in the steady state equation $\mathbf{Y}\mathbf{A}\mathbf{v}=0$.

In a canonical flux regime, i.e. when irreversible reactions have a zero lower bound on reaction rates, all nonstoichiometric BCs are in the type-I category. As their factorization suggests, their emergence relies specifically on the irreversibility patterns and not on the exact values of the flux bounds. This makes the detection of these BCs insensitive to potential modeling errors in the form of inaccuracies in flux bounds, even when they appear in a non-canonical flux regime. However, BCs in this category have the following undesirable property:

#### Proposition 3

Let a network $G$ contain a type-I nonstoichiometric BC. There exist at least two blocked irreversible reactions in the network.

It follows that once all blocked reactions are removed, no type-I nonstoichiometric BCs may exist in the network. In fact, the removal of such reactions changes the linkage structure of the network such that all type-I nonstoichiometric BCs in the original network appear as stoichiometric BCs in the reduced network.

Type-II nonstoichiometric BCs do not automatically require a number of network reactions to be blocked in the steady state flux set. By contrast, they require a number of reactions to be fixated at a nonzero upper- or lower bound, as the following statement demonstrates.

#### Proposition 4

Let $G$ be a network all blocked reactions of which have been removed. Suppose $G$ contains a nonstoichiometric BC. There exist at least three reactions in $G$, which are fixated at a corresponding nonzero lower- or upper bound, for all $\mathbf{v}\in \mathcal{F}\left(G\right)$.

More specifically, it can be shown that the existence of a type-II nonstoichiometric BC implies at least one irreversible reaction is fixated at a positive lower bound. It is not surprising that such BCs only emerge in non-canonical flux regimes. However, an undesirable feature with type-II nonstoichiometric BCs is that their factorization relies explicitly on the values of upper- and lower bounds, which may make their detection sensitive to modeling assumptions.

### Toy examples

Distinct classes of balanced complexes identified in this study are practically observed in all sorts of small and large networks. However, here we present small networks specifically designed for illustrative purposes. The fact that the chemistry in these toy networks may seem unrealistic or may not reflect real-world mechanisms should not be viewed critically; they were constructed with the idea of providing simple illustrations involving very few species.

**Figure 3:**
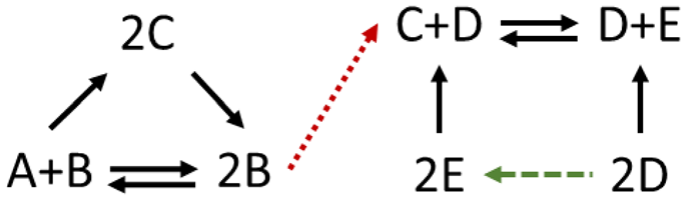
Stoichiometric BCs in a toy network. The conversion diagram depicts a base network with $m=5$ species, $n=7$ complexes, and $r=6$ base reactions, two of which are reversible. The network has four BCs, which will remain balanced even if one adds the dashed reaction (highlighted in green). However, once one adds the dotted reaction (highlighted in red), only two of these complexes remain balanced.

With that in mind, let us next consider the toy network on Fig. 3.

Let us first only consider the base network in Fig. 3, which excludes the dotted and dashed reactions. It is trivial to show that $(A+B)$ and $\left(2B\right)$ have strictly stoichiometric factorizations. The two complexes $(2C)$ and $\left(C+D\right)$ are stoichiometric BCs but not strictly stoichiometric ones. All four complexes will remain balanced, if one changes the irreversibility of reactions arbitrarily or if the dashed reaction is added to the network. However, as soon as one adds the dotted reaction to the network, the linkage structure will be altered. As a result, the strictly stoichiometric BCs $(A+B)$ and $\left(2B\right)$ will still be balanced, but the stoichiometric BCs $(2C)$ and $\left(C+D\right)$ will not remain balanced.

**Figure 4:**
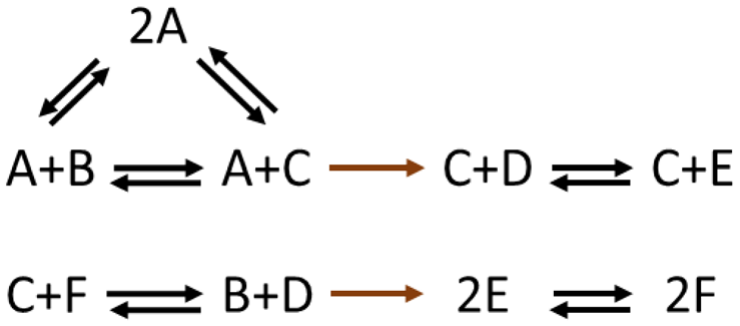
A nonstoichiometric BC in a toy network. The conversion diagram depicts a network with $m=6$ species, $n=9$ complexes, and $r=8$ reactions. The network has one balanced complex ($2A$) which is a type-I nonstoichiometric BC. As predicted by Proposition 3, the two irreversible reactions in this network (highlighted in brown) are both blocked at steady state.

Nonstoichiometric BCs are more complex phenomena that are expected to emerge more frequently in larger networks, and, thus, are more challenging to illustrate in toy networks. To demonstrate an example, let us consider the conversion diagram in Fig. 4.

The network in Fig. 4 contains no stoichiometric BCs. Let us sort the species by alphabetical order and sort the complexes as follows:$\left({C}_{1},\ldots ,{C}_{9}\right)=(A+B,\, A+C,\, C+D, \,C+E, \,C+F, \,B+D,\, 2E,\, 2F,\,2A)$. Given the linkage structure of this network, let us define the basis matrix $\mathbf{U}$ as follows

$${\mathbf{U}} = \left[ {\begin{array}{*{20}c} {1 1 1 1 0 0 0 0 1} \\ {0 0 0 0 1 1 1 1 0} \\ \end{array} } \right]^{T} .$$

It can be shown that the complex indexed by vector ${\mathbf{e}}_{9}$ has a type-I nonstoichiometric factorization with the following parameters

$$\begin{aligned} & {{\varvec{\upzeta}}}_{1} = \left[ {1 0 0 0 0 0} \right]^{T} ,\;\;{{\varvec{\upzeta}}}_{2} = \left[ {1 0 0 1 1 1} \right]^{T} ;\;\;{{\varvec{\upxi}}}_{1} = \left[ { - 1 0} \right]^{T} ,\;\;{{\varvec{\upxi}}}_{2} = \left[ { - 1 - 1} \right]^{T} ; \\ & {{\varvec{\uptheta}}}_{1} = \left[ {0 0 1 1 0 0 0 0 0} \right]^{T} ,\;\;{{\varvec{\uptheta}}}_{2} = \left[ {0 0 0 0 0 0 1 1 0} \right]^{T} ; \\ \end{aligned}$$

It follows that ${c}_{9}$ is a type-I nonstoichiometric BC for the network. It can also be shown that the two irreversible reactions (shown in brown) are blocked at steady state, which is consistent with the prediction of Proposition 3. Note that removing the two blocked reactions would alter the linkage structure of the network, as a result of which the complex ${C}_{9}=2A$ would become a stoichiometric BC for the reduced network.

It is worth noting that changing the irreversibility patterns elsewhere in the network does not affect the balancing property; however, ${C}_{9}$ would not be balanced anymore if one modifies either of the two reactions in brown. To see this fact, consider the counterexample with flux distribution ${v}_{{C}_{1}\to {C}_{2}}=1$, ${v}_{{C}_{2}\to {C}_{3}}=-1$, ${v}_{{C}_{5}\to {C}_{6}}=2$, ${v}_{{C}_{6}\to {C}_{7}}=1$, ${v}_{{C}_{7}\to {C}_{8}}=1$, ${v}_{{C}_{2}\to {C}_{9}}=-1$ and the rest of fluxes equal zero. It is precisely the irreversibility of these two reactions in this particular constellation that makes ${C}_{9}$ a balanced complex.

### Presence of the different categories of BCs in metabolic models

**Figure 5:**
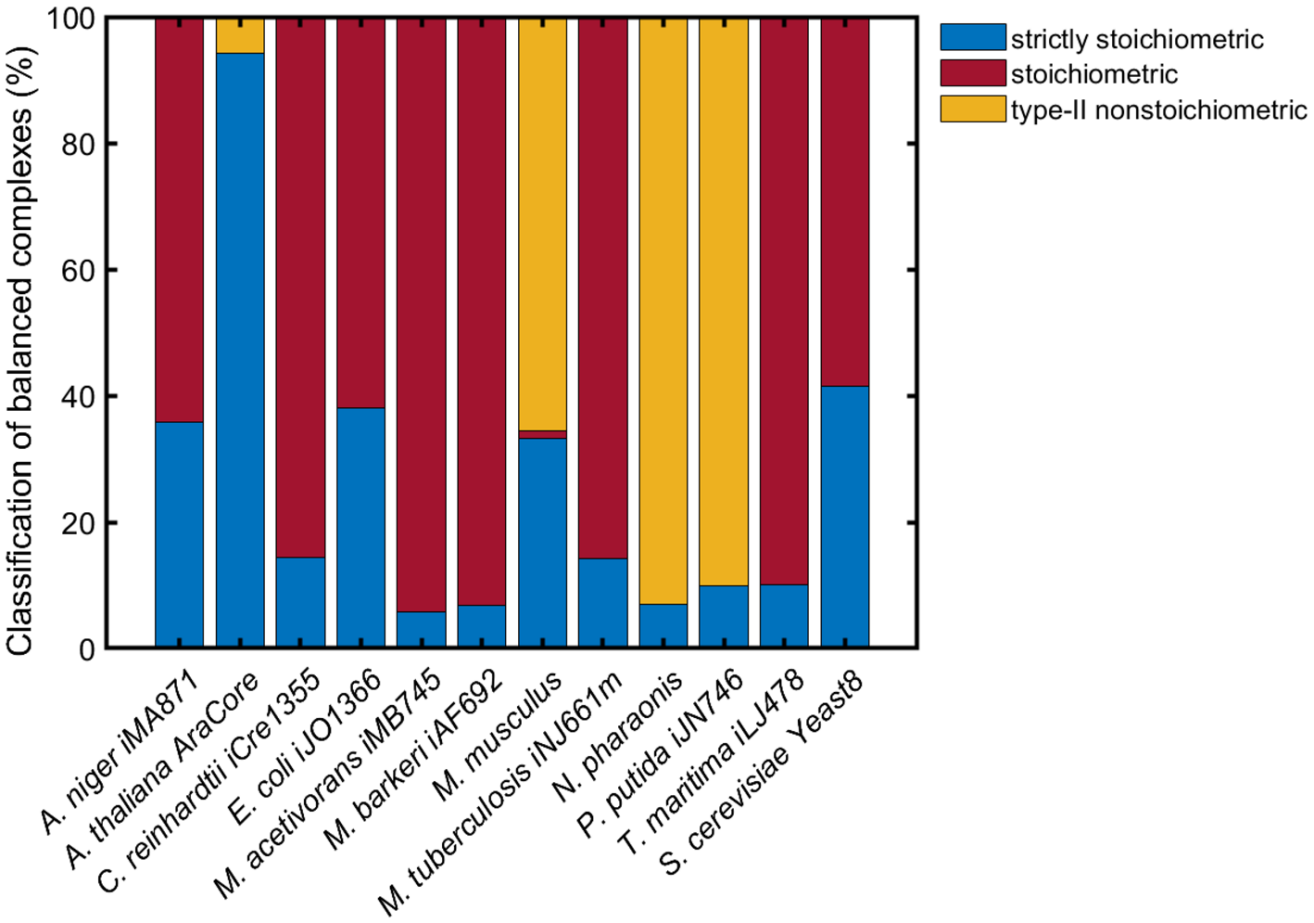
Classification of balanced complexes into strictly stoichiometric, stoichiometric and type-II nonstoichiometric. Balanced complexes from twelve genome-scale models of organisms across kingdoms of life are classified and the percentage of model BCs that fall into the respective class is presented. Type-I nonstoichiometric BCs are not present in the analyzed models since blocked reactions were removed from the networks prior to analyses. For model references, see Supplementary Information.

To show the prevalence of the proposed categorization of BCs in real-world networks, we categorize balanced complexes identified in twelve genome-scale metabolic networks of organisms from all kingdoms of life^12^. While the analyzed networks differ in size, the emergence of balanced complexes is a ubiquitous property for all analyzed networks. However, the percentage of balanced complexes does not correlate with network size (correlation ~ − 0.3 to number of model species, reactions and complexes, respectively, *p*-value > 0.33). The percentage of balanced complexes observed by Küken et al.^12^ was between 1.8% in the model of *N. pharaonis*, followed by 3.2% and 3.8% in metabolic models of *P. putida* and *A. thaliana*, respectively, and reached values up to 43.3% and 58% in models of *M. acetivorans* and *M. barkeri* (Table S1). They removed blocked reactions from the networks prior to the identification of BCs, which results in the absence of type-I nonstoichiometric BCs. Our results indicate that all remaining categories of BCs, namely: strictly stoichiometric, stoichiometric, and type-II nonstoichiometric, are found across all analyzed genome-scale networks. We find that stoichiometric BCs form the largest group of BCs with 59% to 94% of all BCs identified, except for models of *A. thaliana*, *M. musculus*, *N. pharaonis* and *P. putida* where stoichiometric BCs could not be found or comprise below 1.5% of all BCs (Fig. 5). In contrast, the class of strictly stoichiometric BCs is present in all twelve analyzed models, with 6% to 94% (on average 26%) of BCs falling into this category. Type-II nonstoichiometric BCs could be identified in the metabolic models of *A. thaliana* (6%), *M. musculus* (66%), *N. pharaonis* (93%) and *P. putida* (90%). Interestingly, models including type-II nonstoichiometric BCs, were found to include less than 1.5% stoichiometric BCs. Our analysis of metabolic networks from organisms of kingdoms across life shows the prevalence of all proposed factors (stoichiometry, linkage structure and flux bounds) for the formation of BCs in biological networks.

## Concluding remarks

The nonlinearity and dimensionality of steady state equations makes it extremely difficult to study the properties of metabolic networks. While closed-form solutions seem evasive in most cases, recent approaches exploit balanced complexes as a means to reduce metabolic networks and facilitate steady state analysis. However, the underlying mechanisms leading to the formation of balanced complexes have not been studied so far. This work investigates this question and identifies stoichiometry, linkage structure, irreversibility patterns and flux bounds as the main driving factors behind this phenomenon.

The analysis enables us to classify balanced complexes into distinct stoichiometric and nonstoichiometric categories based on their origins and to study their properties. Formulation of the problem as a linear feasibility program provides a computationally efficient tool that is applicable to even large-scale metabolic networks. It is a fascinating aspect of these results that they are not obtained under any specific kinetic assumptions, which suggest they enjoy a ubiquity and are unperturbed by kinetic approximations. Indeed, examination of several well-established metabolic models demonstrates that all these categories are present in distinct metabolic networks across diverse kingdoms of life.

## Supplementary Information

Supplementary Information.

## Data Availability

All networks used in the analysis are available here https://github.com/ankueken/effective_deficiency.

## Code availability

The code is available https://github.com/ankueken/effective_deficiency.
