# Supplementary material for: The unraveling of balanced complexes in metabolic networks
Source: Sci Rep. 2023 Apr 7;13:5712. doi: 10.1038/s41598-023-32666-6 (PMC10082078; doi:10.1038/s41598-023-32666-6)
Supplement: Supplementary file 1 — Supplementary Information. [file 41598_2023_32666_MOESM1_ESM.pdf]

Supplementary Information to

The unraveling of balanced complexes in metabolic networks

Damoun Langary[1,2], Anika Küken[2], Zoran Nikoloski[1,2]

[1]Systems Biology and Mathematical Modeling, Max Planck Institute of Molecular Plant Physiology, Potsdam, Germany
[2]Bioinformatics, Institute of Biochemistry and Biology, University of Potsdam, Potsdam, Germany

S1 Mathematical Analysis of Balanced Complexes

S1.1 Definitions and motivation

Let $G = (\mathcal{S}.\mathcal{C},\mathcal{R})$ be a chemical reaction network with stoichiometric map $\mathbf{Y}$ and complex-to-reaction incidence matrix $\mathbf{A}$. The steady state flux set for G is defined as follows

$$\mathcal{F}(G) = \{\mathbf{v} \mid \mathbf{YAv} = \mathbf{0}\,,\ \ \mathbf{v}_\mathrm{l} \le \mathbf{v} \le \mathbf{v}_\mathrm{u}\}$$

where $\mathbf{v}_\mathrm{l}$ and $\mathbf{v}_\mathrm{u}$ denote lower- and upper bounds for flux through reactions, respectively.

A complex $C_j \in \mathcal{C}$ is referred to as a balanced complex (BC), if for all flux vectors in $\mathcal{F}(G)$ the total flux entering this complex is equal to the total flux leaving this complex. This is equivalent to

$$\mathbf{e}_j{}^\mathrm{T}\mathbf{A}\,\mathbf{v} = \mathbf{0}\ \ , \qquad \forall \mathbf{v} \in \mathcal{F}(G)\,,$$

where $\mathbf{e}_j$ is the vector with a unit value for the jth entry and zero values elsewhere.

For any $R \in \mathcal{R}$, we say R is a blocked reaction in G, if for all the feasible set distributions $\mathbf{v} \in \mathcal{F}(G)$, there is zero flux through R, that is, $v_R = 0$. This is a commonly occurring phenomenon in metabolic networks, especially in scenarios when flux bounds and/or optimization of particular objectives are imposed. Similarly, we say a reaction $R \in \mathcal{R}$ is fixated at some flux value f, if for all flux distributions $\mathbf{v} \in \mathcal{F}(G)$, the flux through R is unchanged, namely, $v_R = f$, $\forall \mathbf{v} \in \mathcal{F}(G)$. Clearly, any blocked reaction is one fixated at zero.

Looking at the stoichiometry of a chemical reaction network, it is easy to see how balanced complexes may emerge. In the most trivial case, any complex comprising a species that appears nowhere else in the network must be balanced at any steady state, thus a BC. Note that this case amounts to finding a single row of the stoichiometric map $\mathbf{Y}$ –as a binary indexing vector for a complex- that defines a balanced complex.

One can readily generalize this idea to come up with more balanced complexes for the network. Suppose a vector $\mathbf{e}_j$ can be expressed as a linear combination of rows of the stoichiometric map $\mathbf{Y}$, that is

$$\exists \boldsymbol{\zeta} :\ \mathbf{e}_j = \mathbf{Y}^\mathrm{T}\boldsymbol{\zeta}\ \ ; \qquad\qquad (1)$$

it immediately follows that

$$\mathbf{e}_j{}^\mathrm{T}\mathbf{A}\,\mathbf{v} = \boldsymbol{\zeta}^\mathrm{T}\mathbf{Y}\,\mathbf{A}\,\mathbf{v} = \mathbf{0}\ \ , \qquad \forall \mathbf{v} \in \mathcal{F}(G)\,.$$

One can further expand the idea by taking into account the left nullspace of the incidence matrix $\mathbf{A}$. Let G be a closed system; the left nullspace of $\mathbf{A}$ has a simplified structure: If the CRN has a connected graph, then the nullspace is the one-dimensional subspace defined as span of basis vector, $\langle\mathbf{1}\rangle$. This yields

$$\exists\ \boldsymbol{\zeta}, \xi\ :\quad \mathbf{e}_j = \mathbf{Y}^{\mathrm{T}}\boldsymbol{\zeta} + \xi\mathbf{1};$$

If the CRN is not connected, then the nullspace has basis vectors of the same structure, but for separate linkage classes. Let us assume G consists of ℓ linkage classes, denoted by $L_1, L_2, \cdots, L_\ell$. With each linkage class l, for $l = 1, \cdots, \ell$, one can associate a vector $\mathbf{u}^{(l)} \in \mathbb{R}^{\mathcal{C}}$ defined as follows

$$u_j^{(l)} = \begin{cases} 0 & C_j \notin L_l \\ 1 & C_j \in L_l \end{cases}.$$

The columns of the matrix $\mathbf{U} = \left[\mathbf{u}^{(1)}\ \mathbf{u}^{(2)}\ \cdots\ \mathbf{u}^{(\ell)}\right] \in \mathbb{R}^{|\mathcal{C}|\times\ell}$ form a basis for the left nullspace of $\mathbf{A}$. Now suppose a vector $\mathbf{e}_j$ can be expressed as follows

$$\exists\ \boldsymbol{\zeta}, \boldsymbol{\xi}\ :\quad \mathbf{e}_j = \mathbf{Y}^{\mathrm{T}}\boldsymbol{\zeta} + \mathbf{U}\,\boldsymbol{\xi}\ ; \qquad (2)$$

it is easy to see that

$$\mathbf{e}_j{}^{\mathrm{T}}\mathbf{A}\,\mathbf{v} = \mathbf{0}\ , \qquad \forall \mathbf{v} \in \mathcal{F}(G)\,.$$

Therefore, $\mathbf{e}_j$ represents a balancing relation for G.

We refer to Eq. (2) as a stoichiometric factorization for the indexing vector $\mathbf{e}_j$. Any BC that can be parameterized in the form of Eq. (2) is referred to as a stoichiometric BC.

Here come the key questions we want to address next: Are these the only forms of balanced complexes that may emerge in a network? In other words, are all balanced complexes in a network simply a result of the stoichiometric- and linkage structure? Could other constraints on a network, such as lower and upper bounds on flux levels, also play a role in creating some other balanced complexes?

S1.2 The primal-dual formulation

To answer these questions, we first formulate the balancing problem as a pair of optimization problems as follows

$$\begin{array}{ll} \text{minimize} & \mathbf{e}_j{}^{\mathrm{T}}\mathbf{A}\,\mathbf{v} \\ \text{subject to} & \begin{array}{l}\mathbf{YA}\,\mathbf{v} = \mathbf{0} \\ \mathbf{v}_l \le \mathbf{v} \le \mathbf{v}_u\end{array} \end{array} \qquad (P1)\quad ,$$

$$\begin{array}{ll} \text{maximize} & \mathbf{e}_j{}^{\mathrm{T}}\mathbf{A}\,\mathbf{v} \\ \text{subject to} & \begin{array}{l}\mathbf{YA}\,\mathbf{v} = \mathbf{0} \\ \mathbf{v}_l \le \mathbf{v} \le \mathbf{v}_u\end{array} \end{array} \qquad .$$

For the problem formulation to conform to standard conventions in convex optimization, we simply replace the 2nd optimization problem by an equivalent minimization problem as follows.

$$\begin{array}{ll} \text{minimize} & -\,\mathbf{e}_j{}^{\mathrm{T}}\mathbf{A}\,\mathbf{v} \\ \text{subject to} & \begin{array}{l}\mathbf{YA}\,\mathbf{v} = \mathbf{0} \\ \mathbf{v}_l \le \mathbf{v} \le \mathbf{v}_u\end{array} \end{array} \qquad (P2)\quad .$$

A vector $\mathbf{e}_j$ represents a balanced complex, if and only if both optimization problems $P1$ & $P2$ have an optimal value of zero.

$$p_1^* = p_2^* = 0\,.$$

Any BC has to satisfy this criterion.

To further analyze which vectors $\mathbf{e}_j$ may satisfy this criterion, we will transform these optimization problems into Lagrange dual forms. It is well-established that strong duality will hold for these convex problems with linear constraints [1], hence the optimal duality gap is zero. Therefore, instead of directly solving $P1$ and $P2$, one can try to solve the dual problems.

To derive the dual problem for $P1$, let us first define the Lagrangian as follows

$$\mathcal{L}(\mathbf{v}, \boldsymbol{\zeta}, \boldsymbol{\lambda}_{\mathrm{u}}, \boldsymbol{\lambda}_{\mathrm{l}}) = {\mathbf{e}_j}^{\mathrm{T}}\mathbf{A}\,\mathbf{v} - \boldsymbol{\zeta}^{\mathrm{T}}(\mathbf{YA}\,\mathbf{v}) + {\boldsymbol{\lambda}_{\mathrm{u}}}^{\mathrm{T}}(\mathbf{v} - \mathbf{v}_{\mathrm{u}}) + {\boldsymbol{\lambda}_{\mathrm{l}}}^{\mathrm{T}}(\mathbf{v}_{\mathrm{l}} - \mathbf{v}) \quad , \qquad \boldsymbol{\lambda}_{\mathrm{u}}, \boldsymbol{\lambda}_{\mathrm{l}} \geq \mathbf{0}\ .$$

The Lagrange dual function is then defined as follows

$$\tilde{g}(\boldsymbol{\zeta}, \boldsymbol{\lambda}) = \inf_{\mathbf{v}} \mathcal{L}(\mathbf{v}, \boldsymbol{\zeta}, \boldsymbol{\lambda}) \quad , \qquad \boldsymbol{\lambda} \stackrel{\mathrm{def}}{=} (\boldsymbol{\lambda}_{\mathrm{u}}, \boldsymbol{\lambda}_{\mathrm{l}})\,.$$

Since the Lagrangian is an affine function of $\mathbf{v}$, it is unbounded from below, hence its infimum value is in general $-\infty$, unless the slope of the affine function is zero, that is

$$\boldsymbol{\nabla}_{\mathbf{v}}\mathcal{L} = \mathbf{A}^{\mathrm{T}}\mathbf{e}_j - \mathbf{A}^{\mathrm{T}}\mathbf{Y}^{\mathrm{T}}\boldsymbol{\zeta} + \boldsymbol{\lambda}_{\mathrm{u}} - \boldsymbol{\lambda}_{\mathrm{l}} \ = \mathbf{0}\ \ ,$$

in which case, the Lagrange dual function would be simplified to the following function

$$g(\boldsymbol{\lambda}) = {\boldsymbol{\lambda}_{\mathrm{l}}}^{\mathrm{T}}\mathbf{v}_{\mathrm{l}} - {\boldsymbol{\lambda}_{\mathrm{u}}}^{\mathrm{T}}\mathbf{v}_{\mathrm{u}}$$

Hence, the dual problem is

$$\begin{array}{ll} \underset{\boldsymbol{\lambda},\boldsymbol{\zeta}}{\text{maximize}} & {\boldsymbol{\lambda}_{\mathrm{l}}}^{\mathrm{T}}\mathbf{v}_{\mathrm{l}} - {\boldsymbol{\lambda}_{\mathrm{u}}}^{\mathrm{T}}\mathbf{v}_{\mathrm{u}} \\ \text{subject to} & \mathbf{A}^{\mathrm{T}}\mathbf{e}_j - \mathbf{A}^{\mathrm{T}}\mathbf{Y}^{\mathrm{T}}\boldsymbol{\zeta} + \boldsymbol{\lambda}_{\mathrm{u}} - \boldsymbol{\lambda}_{\mathrm{l}} = \mathbf{0} \qquad (D1)\ . \\ & \boldsymbol{\lambda}_{\mathrm{u}}, \boldsymbol{\lambda}_{\mathrm{l}} \geq \mathbf{0} \end{array}$$

Similarly, one can process the optimization problem $P2$ to arrive at the following dual formulation

$$\begin{array}{ll} \underset{\boldsymbol{\lambda},\boldsymbol{\zeta}}{\text{maximize}} & {\boldsymbol{\lambda}_{\mathrm{l}}}^{\mathrm{T}}\mathbf{v}_{\mathrm{l}} - {\boldsymbol{\lambda}_{\mathrm{u}}}^{\mathrm{T}}\mathbf{v}_{\mathrm{u}} \\ \text{subject to} & -\mathbf{A}^{\mathrm{T}}\mathbf{e}_j + \mathbf{A}^{\mathrm{T}}\mathbf{Y}^{\mathrm{T}}\boldsymbol{\zeta} + \boldsymbol{\lambda}_{\mathrm{u}} - \boldsymbol{\lambda}_{\mathrm{l}} = \mathbf{0} \qquad (D2)\ . \\ & \boldsymbol{\lambda}_{\mathrm{u}}, \boldsymbol{\lambda}_{\mathrm{l}} \geq \mathbf{0} \end{array}$$

For $\mathbf{e}_j$ to represent a BC, both problems $D1$ and $D2$ must have zero optimal values, i.e.

$$d_1^* = d_2^* = 0\,.$$

It is worth noting that, while the primal problems $P1$ and $P2$ have a slightly different objective but share the same constraints, the dual problems share the same objective function while having a slightly different feasible set.

S1.3 Analyzing the dual problems(s) in a canonical flux regime

S1.3.1 Analyzing the dual objective

The objective function for the dual problems can be written as

$$\tilde{g}(\boldsymbol{\lambda}) = \boldsymbol{\lambda}_{\mathrm{l}}{}^{\mathrm{T}}\mathbf{v}_{\mathrm{l}} - \boldsymbol{\lambda}_{\mathrm{u}}{}^{\mathrm{T}}\mathbf{v}_{\mathrm{u}} = \sum_{R\in\boldsymbol{\mathcal{R}}}\left(\lambda_{\mathrm{l},R}v_{\mathrm{l},R} - \lambda_{\mathrm{u},R}v_{\mathrm{u},R}\right) \quad . \tag{3}$$

To simplify this objective function, let us assume the CRN operates in a *canonical flux regime*, that is the bounds on reaction fluxes are standardized as follows

$$\begin{aligned} &\mathbf{v}_{\mathrm{u}} > \mathbf{0} \ ; \\ &v_{\mathrm{l},R} < 0, \qquad \forall R \in \mathcal{R}^{\mathrm{rev}} \ ; \\ &v_{\mathrm{l},R} = 0, \qquad \forall R \in \mathcal{R}^{\mathrm{irr}} \ ; \end{aligned}$$

where $v_{\mathrm{l},R}$ denotes the Rth entry of vector $\mathbf{v}_{\mathrm{l}}$, corresponding to reaction $R \in \mathcal{R}$. In a canonical regime, the irreversible reactions are exactly those corresponding to zero entries of $\mathbf{v}_{\mathrm{l}}$, while the rest of entries in $\mathbf{v}_{\mathrm{l}}$ must be negative and correspond to reversible reactions.

In a canonical regime, it is easy to verify that all terms on the right-hand side of Eq. (3) are nonpositive, given the dual feasibility conditions $\boldsymbol{\lambda}_{\mathrm{u}}, \boldsymbol{\lambda}_{\mathrm{l}} \geq \mathbf{0}$. For the sum of all those terms to equal zero, every single term in the RHS must be zero. At any optimal point, we must have

$$\begin{aligned} \lambda_{\mathrm{u},R}, v_{\mathrm{u},R} &= 0 \ , \qquad \forall R \in \mathcal{R} \ ; \\ \lambda_{\mathrm{l},R}, v_{\mathrm{l},R} &= 0 \ , \qquad \forall R \in \mathcal{R} \ . \end{aligned}$$

This, in turn, yields

$$\begin{aligned} \lambda_{\mathrm{u},R} &= 0\,, \qquad \forall R \in \mathcal{R} \ ; \\ \lambda_{\mathrm{l},R} &= 0\,, \qquad \forall R \in \mathcal{R}^{\mathrm{rev}} \, . \end{aligned}$$

Therefore, for the complex balancing to hold, out of all entries in $\boldsymbol{\lambda}_{\mathrm{u}}, \boldsymbol{\lambda}_{\mathrm{l}}$, only those associated with lower bounds of irreversible reactions can take arbitrary values (though they must be nonnegative due to dual feasibility). The rest of entries in $\boldsymbol{\lambda}_{\mathrm{u}}, \boldsymbol{\lambda}_{\mathrm{l}}$ must all be zero.

$$\begin{aligned} \boldsymbol{\lambda}_{\mathrm{u}} &= \mathbf{0}\,, \\ \boldsymbol{\lambda}_{\mathrm{l}} &= \boldsymbol{\lambda}_{\mathrm{l}}^{\mathrm{irr}}\,. \end{aligned}$$

Note that $\boldsymbol{\lambda}_{\mathrm{u}}, \boldsymbol{\lambda}_{\mathrm{l}}$ having the above form is a necessary condition for the dual problem(s) to admit feasible solution(s) with an objective value of zero. Moreover, if such a solution is found, then it is clearly an optimal point.

It is also worth noting that under a canonical flux regime, the upper bounds on flux levels (as well as negative lower bounds on flux through reversible reactions) have no impact on the formation of balanced complexes. Hence, as far as the emergence of balanced complexes is concerned, it makes sense to just ignore those upper bounds on fluxes and simply study the resulting steady state flux cone.

Moreover, for some $\mathbf{e}_j$ to represent a BC, the dual variables must also satisfy the other constraints in dual problems $D1$ and $D2$.

$$\mathbf{A}^{\mathrm{T}}\mathbf{e}_j = \mathbf{A}^{\mathrm{T}}\mathbf{Y}^{\mathrm{T}}\boldsymbol{\zeta}_1 + \boldsymbol{\lambda}_{l1}^{\mathrm{irr}} \ , \qquad (4\text{-}1)$$
$$\mathbf{A}^{\mathrm{T}}\mathbf{e}_j = \mathbf{A}^{\mathrm{T}}\mathbf{Y}^{\mathrm{T}}\boldsymbol{\zeta}_2 - \boldsymbol{\lambda}_{l2}^{\mathrm{irr}} \ . \qquad (4\text{-}2)$$

Both the above equations must hold, for the dual problems $D1$ and $D2$ to both have zero optimal values, that is, $d_1^* = d_2^* = 0$.

Therefore, any vector $\mathbf{e}_j$ which satisfies the above two equations for arbitrary parameter values $\boldsymbol{\zeta}_1, \boldsymbol{\zeta}_2$ and $\boldsymbol{\lambda}_{l1}^{\mathrm{irr}}, \boldsymbol{\lambda}_{l2}^{\mathrm{irr}} \geq 0$, provides a zero optimal solution for the primal optimization problem and hence, represents a balanced complex for the given CRN. In other words, this can be seen as a necessary and sufficient condition, under a canonical flux regime.

S1.3.2 Relation to stoichiometric factorization

Let us further study the two equalities in Eq. (4). First of all, both vectors $\boldsymbol{\lambda}_{l1}^{\mathrm{irr}}, \boldsymbol{\lambda}_{l2}^{\mathrm{irr}}$ must lie in the range of $\mathbf{A}^{\mathrm{T}}$, that is, the row space of $\mathbf{A}$. Hence, we can introduce a change of variables

$$\exists \boldsymbol{\theta}_k : \quad \boldsymbol{\lambda}_{lt}^{\mathrm{irr}} = \mathbf{A}^{\mathrm{T}}\boldsymbol{\theta}_t \ , \qquad t = 1,2 \ .$$

Given the discussion in Section S1.3.1, each new variables $\boldsymbol{\theta}_t$ must satisfy the following constraints,

$$\begin{cases} (\mathbf{A}^{:R})^{\mathrm{T}}\boldsymbol{\theta}_t = 0 \ , & \forall R \in \mathcal{R}^{\mathrm{rev}} \\ (\mathbf{A}^{:R})^{\mathrm{T}}\boldsymbol{\theta}_t \geq 0 \ , & \forall R \in \mathcal{R}^{\mathrm{irr}} \end{cases} , \qquad t = 1,2 \ . \qquad (5)$$

Therefore, one can replace the parameters $\boldsymbol{\lambda}_{l1}^{\mathrm{irr}}, \boldsymbol{\lambda}_{l2}^{\mathrm{irr}}$ in Eq. (4) with their equivalents in terms of $\boldsymbol{\theta}_1$ and $\boldsymbol{\theta}_2$, while integrating extra constraints [of dual feasibility] given in Eq. (5).

Using the new parameter $\boldsymbol{\theta}_1$, Eq. (4-1) can be written as

$$\mathbf{A}^{\mathrm{T}}\mathbf{e}_j = \mathbf{A}^{\mathrm{T}}\mathbf{Y}^{\mathrm{T}}\boldsymbol{\zeta}_1 + \mathbf{A}^{\mathrm{T}}\boldsymbol{\theta}_1 \qquad (6\text{-}1) \ ,$$

in which, $\boldsymbol{\zeta}_1$ and $\boldsymbol{\theta}_1$ are independent parameters that are only constrained to satisfy Eq. (5).

The general solution for $\mathbf{e}_j$ in Eq. (6-1) will be of the form

$$\mathbf{e}_j = \langle \text{any particular solution} \rangle + \mathrm{Ker}(\mathbf{A}^T) \ .$$

Assuming the CRN is closed, $\mathrm{Ker}(\mathbf{A}^T)$ has the well-known structure discussed in Section S1.1. Therefore, from Eq. (4), one obtains the following general expressions for $\mathbf{e}_j$

$$\begin{cases} \mathbf{e}_j = \mathbf{Y}^{\mathrm{T}}\boldsymbol{\zeta}_1 + \mathbf{U}\,\boldsymbol{\xi}_1 + \boldsymbol{\theta}_1 & (7\text{-}1) \\ \mathbf{e}_j = \mathbf{Y}^{\mathrm{T}}\boldsymbol{\zeta}_2 + \mathbf{U}\,\boldsymbol{\xi}_2 - \boldsymbol{\theta}_2 & (7\text{-}2) \end{cases} ,$$

for independent parameters $\boldsymbol{\zeta}_1, \boldsymbol{\zeta}_2, \boldsymbol{\xi}_1, \boldsymbol{\xi}_2, \boldsymbol{\theta}_1, \boldsymbol{\theta}_2$, subject to the constraints expressed in Eq. (5).

A special case for $\mathbf{e}_j$ may be obtained by setting $\boldsymbol{\theta}_1 = \boldsymbol{\theta}_2 = 0$. Note that this choice of parameter values also satisfies the constraints in Eq. (5). With $\boldsymbol{\theta}_1 = \boldsymbol{\theta}_2 = 0$, Eq. (7) reduces to two identical equations of the form

$$\mathbf{e}_j = \mathbf{Y}^{\mathrm{T}}\boldsymbol{\zeta}_1 + \mathbf{U}\,\boldsymbol{\xi}_1 \ ,$$

which is exactly the stoichiometric factorization presented in Eq. (2). Therefore, the factorization in Eq. (7), obtained by solving the dual optimization problems, can be viewed as a generalization of the stoichiometric factorization.

In a canonical flux regime, a vector $\mathbf{e}_j$ is said to represent a nonstoichiometric BC, if it has a factorization corresponding to Eq. (7), but no stoichiometric factorization [corresponding to Eq. (2)]. For reasons that will become clear soon, any such a BC is referred to as a *type-I nonstoichiometric BC.*

Now, if one partitions the incidence matrix $\mathbf{A}$ into reversible and irreversible blocks as in $\mathbf{A} = \left[\mathbf{A}^{\text{rev}}\ \mathbf{A}^{\text{irr}}\right]$, then the constraints in Eq. (5) can be rewritten in the following simpler block form

$$\begin{cases} \mathbf{A}^{\text{rev}^{\text{T}}}\boldsymbol{\theta}_k = 0 \\ \mathbf{A}^{\text{irr}^{\text{T}}}\boldsymbol{\theta}_k \geq 0 \end{cases}, \qquad k = 1{,}2\,. \qquad (8)$$

S1.3.3 The factorization of BCs in a canonical flux regime

Assuming the system operates in a canonical flux regime, the discussion above can serve as a proof for the following statement.

Theorem S1. Suppose the network is operating in a canonical flux regime. There exist variables $\boldsymbol{\zeta}_1, \boldsymbol{\zeta}_2 \in \mathbb{R}^{|\mathcal{S}|}$, $\boldsymbol{\xi}_1, \boldsymbol{\xi}_2 \in \mathbb{R}^{\ell}$, $\boldsymbol{\theta}_1, \boldsymbol{\theta}_2 \in \mathbb{R}^{|\mathcal{C}|}$, such that

$$\begin{cases} \mathbf{e}_j = \mathbf{Y}^{\text{T}}\boldsymbol{\zeta}_1 + \mathbf{U}\,\boldsymbol{\xi}_1 + \boldsymbol{\theta}_1 \\ \mathbf{e}_j = \mathbf{Y}^{\text{T}}\boldsymbol{\zeta}_2 + \mathbf{U}\,\boldsymbol{\xi}_2 - \boldsymbol{\theta}_2 \\ \mathbf{A}^{\text{rev}^{\text{T}}}\boldsymbol{\theta}_1 = \mathbf{0} \\ \mathbf{A}^{\text{irr}^{\text{T}}}\boldsymbol{\theta}_1 \geq \mathbf{0} \\ \mathbf{A}^{\text{rev}^{\text{T}}}\boldsymbol{\theta}_2 = \mathbf{0} \\ \mathbf{A}^{\text{irr}^{\text{T}}}\boldsymbol{\theta}_2 \geq \mathbf{0} \end{cases}, \qquad (9)$$

if and only if the complex $C_j \in \mathcal{C}$ is a BC.

Due to the linearity of the above factorizations and constraints, Eq. (9) can serve as a linear feasibility problem, with variables $\boldsymbol{\zeta}_1, \boldsymbol{\zeta}_2, \boldsymbol{\xi}_1, \boldsymbol{\xi}_2, \boldsymbol{\theta}_1, \boldsymbol{\theta}_2$, to check whether any given complex $C_j \in \mathcal{C}$ is a BC of the network. In view of the highly efficient computational tools available for LP problems, this provides an alternative approach for finding balanced complexes. What comes on top of it as a bonus is the fact that it allows one to systematically identify the underlying factors contributing to the formation of any BC.

Let us further reflect on these constraints. Out of all variables in the factorization, only $\boldsymbol{\theta}_1, \boldsymbol{\theta}_2$ are constrained by the following constraints, and $\boldsymbol{\zeta}_1, \boldsymbol{\zeta}_2, \boldsymbol{\xi}_1, \boldsymbol{\xi}_2$ are not. Hence, one may transform the constraints in Eq. (9) into the following -more compact- form

$$\begin{cases} \mathbf{A}^{\text{rev}^{\text{T}}}\left(\mathbf{e}_j - \mathbf{Y}^{\text{T}}\boldsymbol{\zeta}_1 - \mathbf{U}\,\boldsymbol{\xi}_1\right) = 0 \\ \mathbf{A}^{\text{irr}^{\text{T}}}\left(\mathbf{e}_j - \mathbf{Y}^{\text{T}}\boldsymbol{\zeta}_1 - \mathbf{U}\,\boldsymbol{\xi}_1\right) \geq 0 \\ \mathbf{A}^{\text{rev}^{\text{T}}}\left(\mathbf{Y}^{\text{T}}\boldsymbol{\zeta}_2 + \mathbf{U}\,\boldsymbol{\xi}_2 - \mathbf{e}_j\right) = 0 \\ \mathbf{A}^{\text{irr}^{\text{T}}}\left(\mathbf{Y}^{\text{T}}\boldsymbol{\zeta}_2 + \mathbf{U}\,\boldsymbol{\xi}_2 - \mathbf{e}_j\right) \geq 0 \end{cases}, \qquad (10)$$

which can replace Eq. (9) in the LP framework. This may be more practical in terms of implementation, but not necessarily in terms of clarification, because it fails to provide an explicit factorization of $\mathbf{e}_j$, unlike Eq. (9).

S1.3.4 Formation of type-I nonstoichiometric BCs

The following statement sheds light on the properties on type-I nonstoichiometric BCs and how they are formed.

Proposition S2. Let a network G contain a type-I nonstoichiometric BC. There exist at least two blocked irreversible reactions in the network.

<u>Proof</u>: Let $\mathbf{e}_j$ represent the type-I nonstoichiometric BC. It immediately follows from the definition that we must have

$$\boldsymbol{\theta}_1 \neq \mathbf{0}, \qquad \boldsymbol{\theta}_2 \neq \mathbf{0} \ , \tag{11-1}$$

as well as,

$$\boldsymbol{\lambda}_{\mathrm{l}t}^{\mathrm{irr}} = \mathbf{A}^{\mathrm{T}}\boldsymbol{\theta}_t \neq \mathbf{0}\,, \qquad t = 1,2\ . \tag{11-2}$$

The balancing relation itself implies that

$$\mathbf{e}_j{}^{\mathrm{T}}\mathbf{A}\,\mathbf{v} = \mathbf{0}\ , \qquad \forall \mathbf{v} \in \mathcal{F}(G)\,.$$

Replacing $\mathbf{e}_j$ by its factorization equations yields

$$\boldsymbol{\theta}_t{}^{\mathrm{T}}\mathbf{A}\,\mathbf{v} = \mathbf{0}\ , \qquad t = 1,2, \qquad \forall \mathbf{v} \in \mathcal{F}(G)\,,$$

that is,

$$\begin{matrix} \boldsymbol{\lambda}_{\mathrm{l}1}^{\mathrm{irr}\,\mathrm{T}}\ \mathbf{v} = \mathbf{0} \\ \boldsymbol{\lambda}_{\mathrm{l}2}^{\mathrm{irr}\,\mathrm{T}}\ \mathbf{v} = \mathbf{0} \end{matrix}\ , \qquad \forall \mathbf{v} \in \mathcal{F}(G)\,. \tag{12}$$

Note that

$$\boldsymbol{\lambda}_{\mathrm{l}t}^{\mathrm{irr}\,\mathrm{T}}\ \mathbf{v} = \sum_{R\in\mathcal{R}^{\mathrm{irr}}} \lambda_{\mathrm{l}t,R}\ v_R\ , \qquad t = 1,2\,.$$

Having the abovementioned structure of the dual variables $\boldsymbol{\lambda}_{\mathrm{l}t}^{\mathrm{irr}}$, $t = 1,2$, and knowing that $v_R \geq 0$, $\forall R \in \mathcal{R}^{\mathrm{irr}}$, it follows from Eq. (12) that

$$\lambda_{\mathrm{l}t,R}\ v_R = 0\,, \qquad t = 1,2, \qquad \forall R \in \mathcal{R}^{\mathrm{irr}}\,.$$

Taking into consideration that Eq. (11-2) implies $\boldsymbol{\lambda}_{\mathrm{l}t}^{\mathrm{irr}}$ has at least one positive entry for both $t = 1,2$, it follows that for $\mathbf{e}_j$ to represent a nonstoichiometric BC, at least one irreversible reaction must be blocked.

Moreover, the two vectors $\boldsymbol{\lambda}_{\mathrm{l}t}^{\mathrm{irr}}$, $t = 1,2$ cannot be collinear; otherwise, one can cancel out those terms in Eq. (4), which yields a stoichiometric factorization for the BC, which would be a contradiction. Therefore, there exist at least two blocked reactions in the network.■

Corollary S3. Let G be a network operating under a canonical flux regime, all blocked reactions of which have been removed. Then all balanced complexes of G have stoichiometric factorizations.

S1.4 Analyzing the dual problem(s) for general flux bounds

S1.4.1 Derivation of an equivalent LP

Next, we consider the network under general -not necessarily canonical- flux bounds, in which some irreversible reactions may also take positive lower bounds on flux levels. Irrespective of the flux bounds, for a vector $\mathbf{e}_j$ to represent a BC, the dual optimization problems $D1$ and $D2$ must take zero optimal values.

$$g(\boldsymbol{\lambda}) = {\boldsymbol{\lambda}_{\mathrm{l}t}}^{\mathrm{T}}\mathbf{v}_{\mathrm{l}} - {\boldsymbol{\lambda}_{\mathrm{u}t}}^{\mathrm{T}}\mathbf{v}_{\mathrm{u}} = 0 \ , \qquad t = 1,2.$$

Here is what contrast this case from the canonical flux regime: Some terms in the dual objective may now take positive values; hence we are not in a position to further simplify the objective function by asserting that every term on the RHS has to be zero, as we did in Section S1.3.1.

A complex represented by vector $\mathbf{e}_j$ is balanced if and only if both dual problems $D1$ and $D2$ have zero optimal values. Now, we represent the equivalent of Theorem S1 for the more general case:

Theorem S4. A complex $C_j \in \mathcal{C}$ is a BC, if and only if there exist variables $\boldsymbol{\zeta}_1, \boldsymbol{\zeta}_2 \in \mathbb{R}^{|\mathcal{S}|}$, $\boldsymbol{\lambda}_{\mathrm{u}1}, \boldsymbol{\lambda}_{\mathrm{l}1}, \boldsymbol{\lambda}_{\mathrm{u}2}, \boldsymbol{\lambda}_{\mathrm{l}2} \in \mathbb{R}^{|\mathcal{R}|}$ such that

$$\begin{cases} \mathbf{A}^{\mathrm{T}}\mathbf{e}_j - \mathbf{A}^{\mathrm{T}}\mathbf{Y}^{\mathrm{T}}\boldsymbol{\zeta}_1 + \boldsymbol{\lambda}_{\mathrm{u}1} - \boldsymbol{\lambda}_{\mathrm{l}1} = \mathbf{0} \\ {\mathbf{v}_l}^{\mathrm{T}}\boldsymbol{\lambda}_{\mathrm{l}1} - {\mathbf{v}_u}^{\mathrm{T}}\boldsymbol{\lambda}_{\mathrm{u}1} = 0 \\ -\mathbf{A}^{\mathrm{T}}\mathbf{e}_j + \mathbf{A}^{\mathrm{T}}\mathbf{Y}^{\mathrm{T}}\boldsymbol{\zeta}_2 + \boldsymbol{\lambda}_{\mathrm{u}2} - \boldsymbol{\lambda}_{\mathrm{l}2} = \mathbf{0} \\ {\mathbf{v}_l}^{\mathrm{T}}\boldsymbol{\lambda}_{\mathrm{l}2} - {\mathbf{v}_u}^{\mathrm{T}}\boldsymbol{\lambda}_{\mathrm{u}2} = 0 \\ \boldsymbol{\lambda}_{\mathrm{u}1}\,, \boldsymbol{\lambda}_{\mathrm{l}1} \geq \mathbf{0} \\ \boldsymbol{\lambda}_{\mathrm{u}2}\,, \boldsymbol{\lambda}_{\mathrm{l}2} \geq \mathbf{0} \end{cases} . \qquad (13)$$

<u>Proof</u>: let us momentarily take a break from directly addressing optimality conditions for $D1$ and $D2$, and instead try to seek feasible variables that set the corresponding dual objectives to zero. Hence, we now seek variables $\boldsymbol{\zeta}_1, \boldsymbol{\zeta}_2 \in \mathbb{R}^{|\mathcal{S}|}$, $\boldsymbol{\lambda}_{\mathrm{u}1}, \boldsymbol{\lambda}_{\mathrm{l}1}, \boldsymbol{\lambda}_{\mathrm{u}2}, \boldsymbol{\lambda}_{\mathrm{l}2} \in \mathbb{R}^{|\mathcal{R}|}$ that satisfy Eq. (13).

Note that $(\boldsymbol{\zeta}_1, \boldsymbol{\lambda}_{\mathrm{u}1}, \boldsymbol{\lambda}_{\mathrm{l}1})$ gives a feasible point for $D1$, while $(\boldsymbol{\zeta}_2, \boldsymbol{\lambda}_{\mathrm{u}2}, \boldsymbol{\lambda}_{\mathrm{l}2})$ gives a feasible point for $D2$. Therefore, if one denotes the optimal (maximal) values of $D1$ and $D2$ by d_1^*, d_2^*, respectively, the feasibility of these points implies that

$$d_1^* \geq 0\,, \qquad d_2^* \geq 0\,. \qquad (14)$$

On the other hand, p_1^* is the minimum of some function $({\mathbf{e}_j}^{\mathbf{T}}\mathbf{A}\mathbf{v})$ on the steady state flux set, while $-p_2^*$ is the maximum of the same function on the same set. Hence, $p_1^* \leq -p_2^*$. Therefore,

$$p_1^* + p_2^* \leq 0 \ .$$

As a result, the general properties of the duality [1] ensure that

$$d_1^* + d_2^* \leq p_1^* + p_2^* \leq 0 \ . \qquad (15)$$

Taking into account Eqs. (13) and (15), it follows that

$$d_1^* = d_2^* = 0\,.$$

Therefore, even though we did not explicitly enforce optimality in Eq. (13), by the virtue of Eq. (5), any set of variables that satisfies Eq. (13) automatically provides optimality, hence it translates to solutions for the dual optimization problems $D1$ and $D2$, with an achieved optimal value of zero.

On the other hand, if vector $\mathbf{e}_j$ represents a BC, it has to lead to zero optimal values for the dual problem(s), hence there exist variables $\boldsymbol{\zeta}_1, \boldsymbol{\zeta}_2 \in \mathbb{R}^{|\mathcal{S}|}$, $\boldsymbol{\lambda}_{\mathrm{u1}}, \boldsymbol{\lambda}_{\mathrm{l1}}, \boldsymbol{\lambda}_{\mathrm{u2}}, \boldsymbol{\lambda}_{\mathrm{l2}} \in \mathbb{R}^{|\mathcal{R}|}$that satisfy Eq. (13). ■

As a result, in the general –not necessarily canonical- case, Eq. (13) replaces the duality problems, and may serve as a linear feasibility problem to check whether a complex $\mathbf{e}_j$ is a BC.

S1.4.2 Nonstoichiometric balancing with general boundary constraints

Given the two equalities containing $\mathbf{e}_j$ in Eq. (13), it follows that the terms $\boldsymbol{\lambda}_{\mathrm{u1}} - \boldsymbol{\lambda}_{\mathrm{l1}}$ and $\boldsymbol{\lambda}_{\mathrm{u2}} - \boldsymbol{\lambda}_{\mathrm{l2}}$ must lie in the rowspan of $\mathbf{A}$; therefore, similar to Eq. (7), one can introduce new variables $\boldsymbol{\theta}_1$ and $\boldsymbol{\theta}_2$, as follows

$$\boldsymbol{\lambda}_{\mathrm{l1}} - \boldsymbol{\lambda}_{\mathrm{u1}} = \mathbf{A}^{\mathrm{T}}\boldsymbol{\theta}_1\,,$$
$$\boldsymbol{\lambda}_{\mathrm{l2}} - \boldsymbol{\lambda}_{\mathrm{u2}} = \mathbf{A}^{\mathrm{T}}\boldsymbol{\theta}_2\,.$$

Using new variables $\boldsymbol{\theta}_1$ and $\boldsymbol{\theta}_2$, one would arrive at the same factorization structure as in Eq. (7) for the balanced complex $\mathbf{e}_j$, which could pave the way for labeling of $\mathbf{e}_j$ as either a stoichiometric or nonstoichiometric balancing relation. However, note that the other constraints in Eq. (9) do not hold generally, under non-canonical flux regimes. In fact, the solution set of Eq. (9) is a subset of the solution set of Eq. (13), for which those additional constraints also hold.

Instead of using new variables $\boldsymbol{\theta}_1$ and $\boldsymbol{\theta}_2$ to identify nonstoichiometric BC, one can resort to the following equivalent definition: A vector $\mathbf{e}_j$ represents a *nonstoichiometric BC*, if it has an implicit factorization corresponding to Eq. (13), but no factorization of the form Eq. (2).

As we have seen with Proposition S2 and Corollary S3, blocked irreversible reactions play a key role in the formation of (type-I) nonstoichiometric BCs in a canonical flux regime. It follows that they can play exactly the same role in networks operating under general flux bounds. However, one might ask the question: Can other factors also lead to the formation of BCs under general –not necessarily canonical- flux bounds?

To answer this question, let us suppose all blocked reactions have been removed from the network. It follows from Corollary S3 that no type-I nonstoichiometric BC may exist in this network. We aim to investigate whether possibly different forms of nonstoichiometric BCs may emerge in this network. Let us define $\mathcal{R}^{\mathrm{pos}} \subseteq \mathcal{R}^{\mathrm{irr}}$ as the set of irreversible reactions with positive lower bounds on flux levels. Let us now assume there exists a nonstoichiometric BC in this network, represented by vector $\mathbf{e}_j$. From the definition of a nonstoichiometric BC, it follows that

$$\boldsymbol{\lambda}_{\mathrm{l1}} - \boldsymbol{\lambda}_{\mathrm{u1}} \neq 0\,,$$
$$\boldsymbol{\lambda}_{\mathrm{l2}} - \boldsymbol{\lambda}_{\mathrm{u2}} \neq 0\,.$$

Moreover, it can be shown that the above two vectors cannot be collinear. Otherwise the equality

$$(\boldsymbol{\lambda}_{l1} - \boldsymbol{\lambda}_{u1}) + (\boldsymbol{\lambda}_{l2} - \boldsymbol{\lambda}_{u2}) = \mathbf{A}^{T}\mathbf{Y}^{T}(\boldsymbol{\zeta}_2 - \boldsymbol{\zeta}_1)\,,$$

would mean that both $(\boldsymbol{\lambda}_{lt} - \boldsymbol{\lambda}_{ut}),\ t = 1{,}2$ can be replaced by a scalar factor of $\mathbf{A}^{T}\mathbf{Y}^{T}(\boldsymbol{\zeta}_2 - \boldsymbol{\zeta}_1)$, hence $\mathbf{e}_j$ would have a stoichiometric factorization, which is a contradiction. Therefore, the two vectors $(\boldsymbol{\lambda}_{lt} - \boldsymbol{\lambda}_{ut}),\ t = 1{,}2$ are linearly independent. The other constraints in Eq. (13),

$$\begin{matrix} \boldsymbol{\lambda}_{lt}{}^{T}\mathbf{v}_l - \boldsymbol{\lambda}_{ut}{}^{T}\mathbf{v}_u = 0\,, \\ \boldsymbol{\lambda}_{ut}\,, \boldsymbol{\lambda}_{lt} \geq \mathbf{0}\,, \end{matrix} \qquad t = 1{,}2\ ,$$

alongside $\mathbf{v}_u > \mathbf{0}$ imply that $\boldsymbol{\lambda}_{ut}{}^{T}\mathbf{v}_u \geq 0,\ \boldsymbol{\lambda}_{lt}{}^{T}\mathbf{v}_l \geq 0\,,\ t = 1{,}2$; and we must also have

$$\begin{matrix} \boldsymbol{\lambda}_{l1} \neq 0\,, \\ \boldsymbol{\lambda}_{l2} \neq 0\,. \end{matrix}$$

In addition, it is easy to show [by contradiction]

$$\exists R_1, R_2 \in \mathcal{R}^{\text{pos}}\ :\ \lambda_{l1,R_1} > 0\ ,\ \lambda_{l2,R_2} > 0\,, \qquad (16)$$ [i]

in which, $\mathcal{R}^{\text{pos}}$ denotes the set of (irreversible) reactions with a positive flux lower bound.

From the definition of the balanced complex, we know that $\mathbf{e}_j{}^{T}\mathbf{A}\,\mathbf{v} = \mathbf{0}\ ,\ \forall \mathbf{v} \in \mathcal{F}(G)$. Replacing $\mathbf{e}_j{}^{T}\mathbf{A} = \left(\mathbf{A}^{T}\mathbf{e}_j\right)^{T}$ by its equivalent from Eq. (13) then yields

$$(\boldsymbol{\lambda}_{ut} - \boldsymbol{\lambda}_{lt})^{T}\,\mathbf{v} = \mathbf{0}\ , \qquad \forall \mathbf{v} \in \mathcal{F}(G)\,, \qquad t = 1{,}2\,. \qquad (17)$$

Therefore, existence of a nonstoichiometric balancing relation in the network implies the existence of at least two independent linear couplings of reaction fluxes.

In addition, via combining Eq. (17) with Eqs. (13-2) and (13-4), one obtains

$$\boldsymbol{\lambda}_{ut}{}^{T}(\mathbf{v} - \mathbf{v}_u) + \boldsymbol{\lambda}_{lt}{}^{T}(\mathbf{v}_l - \mathbf{v})\ = \mathbf{0}\ , \qquad \forall \mathbf{v} \in \mathcal{F}(G)\,, \qquad t = 1{,}2\,.$$

However, we know that

$$\boldsymbol{\lambda}_u \geq \mathbf{0}\,, \qquad \mathbf{v} - \mathbf{v}_u \leq \mathbf{0}\,, \qquad \boldsymbol{\lambda}_l \geq \mathbf{0}\,, \qquad \mathbf{v}_l - \mathbf{v} \geq \mathbf{0}\,,$$

Therefore, all reactions for which at least one corresponding entry of $\boldsymbol{\lambda}_{u1}, \boldsymbol{\lambda}_{u2}, \boldsymbol{\lambda}_{l1}, \boldsymbol{\lambda}_{l2}$ is nonzero, must be fixated at their respective lower/upper bounds for all values of $\mathbf{v}$ in the steady state flux set. In particular, Eq. (16) ensures that for at least one irreversible reaction with a positive lower bound is fixated at the minimum for all $\mathbf{v} \in \mathcal{F}(G)$.

Proposition S5. Let G be a network all blocked reactions of which have been removed. Suppose G contains a nonstoichiometric BC. There exists at least one irreversible reaction with a positive lower bound $R \in \mathcal{R}^{\text{pos}}$, such that $v_R = v_{l,R}\ ,\ \forall \mathbf{v} \in \mathcal{F}(G)$.

The following statement follows immediately from Proposition S5.

---

[i] Otherwise, it can be shown that some irreversible reactions must be blocked.

Proposition S6. Let G be a network all blocked reactions of which have been removed. Suppose G contains a nonstoichiometric BC. There exist at least three reactions in G , which are fixated at a corresponding [nonzero] lower- or upper bound, for all $\mathbf{v} \in \mathcal{F}(G)$.

Proof: From Eq. (16), we know that $\exists R_1 \in \mathcal{R}^{\text{pos}} : \lambda_{\text{l}1,R_1} > 0$, and the reaction R_1 is fixated at the corresponding lower bound. From the BC constraints in Eq. (17), we have $\boldsymbol{\lambda}_{\text{l}1}{}^{\text{T}}\mathbf{v}_{\text{l}} - \boldsymbol{\lambda}_{\text{u}1}{}^{\text{T}}\mathbf{v}_{\text{u}} = \lambda_{\text{l}1,R_1} v_{\text{l},R_1} + \sum_{\rho \neq R_1} \lambda_{\text{l}1,\rho} v_{\text{l},\rho} - \sum_{\rho} \lambda_{\text{u}1,\rho} v_{\text{u},\rho} = 0$. Since $\lambda_{\text{l}1,R_1} v_{\text{l},R_1} > 0$, it follows that

$$\exists R_1' \neq R_1 \colon \lambda_{\text{l}1,R_1'} > 0\,, v_{\text{l},R_1'} < 0 \qquad \text{or} \qquad \exists R_1' \neq R_1 \colon \lambda_{\text{u}1,R_1'} > 0\,, v_{\text{u},R_1'} > 0\ .$$

Therefore, there exist at least two distinct reactions $R_1 \in \mathcal{R}^{\text{pos}}, R_1' \in \mathcal{R}$ fixated at their corresponding flux bounds.

With the same logic, the other constraint $\boldsymbol{\lambda}_{\text{l}2}{}^{\text{T}}\mathbf{v}_{\text{l}} - \boldsymbol{\lambda}_{\text{u}2}{}^{\text{T}}\mathbf{v}_{\text{u}} = 0$ implies that there exist at least two distinct reactions $R_2 \in \mathcal{R}^{\text{pos}}, R_2' \in \mathcal{R}$ fixated at their corresponding flux bounds. Now, if there exist any other reaction $R_3 \notin \{R_1, R_1', R_2, R_2'\}$ for which the strict inequality $\lambda_{\text{l}t,R_3} + \lambda_{\text{u}t,R_3} > 0$ holds for some $t \in \{1,2\}$, then we have found the three fixated reactions and the proof is complete.

If that is not the case, then we cannot have both $R_1 = R_2$ and $R_1' = R_2'$; otherwise, the two vectors $(\boldsymbol{\lambda}_{\text{l}1} - \boldsymbol{\lambda}_{\text{u}1}), (\boldsymbol{\lambda}_{\text{l}2} - \boldsymbol{\lambda}_{\text{u}2})$ would become collinear, which is a contradiction. Therefore, there exist at least three reactions fixated at nonzero upper- or lower bounds.■

S1.4.3 Type-I vs. type-II nonstoichiometric BCs

Let us remind that in networks operating under a canonical flux regime, any BC encoded by vector $\mathbf{e}_j$ must satisfy the factorization scheme presented in Eq. (7). We showed that for a nonstoichiometric BC to exist, at least two irreversible reactions must be blocked at steady state. It can also be shown that those blocked reactions cannot lie within any strong linkage class of the network, but each must be a "bridge" reaction that connects distinct strong linkage classes within a linkage class[i]. We refer to this class of nonstoichiometric BCs as *type-I*.

It is not surprising that type-I nonstoichiometric BCs can also emerge in non-canonical flux regimes. To seek type-I nonstoichiometric BCs under general flux bounds, one can basically apply the same formulation as Eq. (9). There is, however, a minor caveat due to the potential existence of positive lower bounds on fluxes. Eq. (9) was introduced in the context of canonical flux bounds, where $\mathbf{A}^{\text{irr}}$ denoted exactly those columns of $\mathbf{A}$ corresponding to zero lower bounds in $\mathbf{v}_{\text{l}}$, and $\mathbf{A}^{\text{rev}}$ was its complementary block in $\mathbf{A}$. To stay consistent with that convention in more general case, the columns corresponding to positive lower bounds need to be removed from $\mathbf{A}^{\text{irr}}$, and instead transferred to $\mathbf{A}^{\text{rev}}$.[ii] Thereby, $\mathbf{A}^{\text{irr}}$ corresponds to $\mathcal{R}^{\text{irr}} \setminus \mathcal{R}^{\text{pos}}$ and $\mathbf{A}^{\text{rev}}$ corresponds to $\mathcal{R}^{\text{rev}} \cup \mathcal{R}^{\text{pos}}$. Whenever we speak of a factorization

---

[i] We showed that $\boldsymbol{\lambda}_{\text{l}}^{\text{irr}} \geq 0$ must be of the form $\boldsymbol{\lambda}_{\text{l}}^{\text{irr}} = \mathbf{A}^{\text{T}}\boldsymbol{\theta}$. It follows that he algebraic sum of the entries in $\boldsymbol{\lambda}_{\text{l}}^{\text{irr}}$ corresponding to any reaction cycle (loop in the graph) must be zero. Taking into account also the fact that $\boldsymbol{\lambda}_{\text{l}}^{\text{irr}} \geq \mathbf{0}$ and $\boldsymbol{\lambda}_{\text{l}}^{\text{rev}} = \mathbf{0}$, it follows that every entry of $\boldsymbol{\lambda}_{\text{l}}^{\text{irr}}$ corresponding to a reaction inside a strong linkage class must be zero.

[ii] This replaces the original partitioning $\mathbf{A} = [\mathbf{A}^{\text{rev}}\ \ \mathbf{A}^{\text{irr}}]$ into $\mathbf{A} = [\mathbf{A}^{\text{zr}}\ \ \mathbf{A}^{\text{nz}}]$, where $\mathbf{A}^{\text{zr}}$ denotes the columns corresponding to (irreversible) reactions with zero lower bounds.

of the form Eq. (9) in the context of networks operating under general flux bounds, we also mean to incorporate this minor discretion.

The obtained factorization scheme demonstrates an interesting property of type-I nonstoichiometric BCs: the emergence of such BCs does not rely on the value of the flux bounds (specified by $\mathbf{v}_\mathrm{l}$, $\mathbf{v}_\mathrm{u}$). Consequently, BCs of this type originate from the network topology, and in particular, the existing irreversibility patterns. It follows that potential modeling errors in the form of inaccuracies in determining upper- and lower-bounds on fluxes has no impact on our ability to detect such BCs in a given network.

What is less desirable about them is the fact that they emerge in conjunction with blocked reactions in the network. Alternatively, suppose we first remove those blocked "bridge" reactions from the network. This would break down some linkage classes of the network into a number of smaller linkage classes, which means we would come up with a reduced network of slightly different linkage structure. This, in turn, expands the left nullspace of the incidence matrix $\mathbf{A}$, that is, the matrix $\mathbf{U}$ will contain a higher number of columns, i.e., basis vectors. It follows that the stoichiometric factorization,

$$\exists\ \boldsymbol{\zeta}, \boldsymbol{\xi}\ :\quad \mathbf{e}_j = \mathbf{Y}^\mathrm{T}\boldsymbol{\zeta} + \mathbf{U}\,\boldsymbol{\xi}\ ,$$

may have a larger solution set in the case of the reduced network. This means, the reduced network obtained by the removal of blocked reactions may accommodate a higher number of stoichiometric BCs. In fact, one can show that all type-I nonstoichiometric BCs of the original network appear as stoichiometric BCs in the reduced network.

A somewhat different phenomenon is the case of nonstoichiometric BCs that may only arise under non-canonical flux regimes, that is, when at least one irreversible reaction has a positive lower bound. We hereby refer to such nonstoichiometric BCs as *type-II*. As was shown in Section S1.4.1, all BCs must satisfy the implicit factorization given in Eq. (13). Assuming all blocked reactions have been removed from some network G, any remaining nonstoichiometric BCs in G must be of type-II. Clearly, the existence of type-II nonstoichiometric BCs in G relies on having a nonempty set $\mathcal{R}^\mathrm{pos}$.

Even though type-II nonstoichiometric BCs emerge in conjunction with the fixation of some reaction fluxes at upper- or lower bounds, as was shown in Section S1.4.2, they do not automatically rely on the network having some blocked reactions that effectively change its linkage structure. This is an interesting property of type-II nonstoichiometric BCs, because of which they can be seen as an unexpected nontrivial phenomenon in the steady state analysis, by contrast to type-I nonstoichiometric BCs, which we associated with unsophisticated network reductions.

Looking back at Eq. (13), the potential existence of a type-II nonstoichiometric BC encoded by $\mathbf{e}_j$ directly relies on the existence of feasible dual variables $\boldsymbol{\lambda}_{\mathrm{u}t}, \boldsymbol{\lambda}_{\mathrm{l}t}$, $t = 1,2$ that fit into the pattern $\boldsymbol{\lambda}_{\mathrm{l}t} - \boldsymbol{\lambda}_{\mathrm{u}t} \in \mathrm{im}(\mathbf{A}^\mathrm{T})$, $t = 1,2$. However, $\boldsymbol{\lambda}_{\mathrm{l}t}, \boldsymbol{\lambda}_{\mathrm{u}t}$, $t = 1,2$ are not free variables, but are tied to each other via other equations in Eq. (13), that is

$$\mathbf{v}_\mathrm{l}^\mathrm{T}\boldsymbol{\lambda}_{\mathrm{l}1} - \mathbf{v}_\mathrm{u}^\mathrm{T}\boldsymbol{\lambda}_{\mathrm{u}1} = 0\ ,$$
$$\mathbf{v}_\mathrm{l}^\mathrm{T}\boldsymbol{\lambda}_{\mathrm{l}2} - \mathbf{v}_\mathrm{u}^\mathrm{T}\boldsymbol{\lambda}_{\mathrm{u}2} = 0\ .$$

This casts light on an undesirable property of type-II nonstoichiometric BCs: the values $\mathbf{v}_\mathrm{l}, \mathbf{v}_\mathrm{u}$ appear explicitly in their factorizations. It follows that potential modeling errors in the form of inaccuracies in determining upper- and lower-bounds on fluxes may have a negative impact and result in a failure to correctly detect type-II nonstoichiometric BCs: if the value of some upper/lower bounds in the model ($\mathbf{v}_\mathrm{l}, \mathbf{v}_\mathrm{u}$) are modified or miscalculated, this could change the values of $\boldsymbol{\lambda}_{\mathrm{l}1} - \boldsymbol{\lambda}_{\mathrm{u}1}$ and $\boldsymbol{\lambda}_{\mathrm{l}2} - \boldsymbol{\lambda}_{\mathrm{u}2}$, which could means they would not necessarily satisfy the factorization for a given $\mathbf{e}_j$ anymore. This sensitivity

of detection to the values of upper- and lower-bounds is an unwelcome feature with type-II nonstoichiometric BCs.

S2 Analysis of biological networks

We categorized balanced complexes identified by Küken et al. [2] in twelve genome-scale metabolic models from organisms of all kingdoms of life (Table S1), namely *A. niger* [3], *A. thaliana* [4], *C. reinhardtii* [5], *E. coli* [6], *M. acetivorans* [7], *M. barkeri* [8], *M. musculus* [9], *M. tuberculosis* [10], *N. pharaonis* [11], *P. putida* [12], *T. maritima* [13] and *S. cerevisiae* [14].

Balanced complexes were identified in networks including no blocked reactions by sequentially solving *P1* and *P2* from Section S1.2 for each complex in a metabolic network. Since blocked reactions are removed these networks do not include type-I nonstoichiometric BCs. To check if a BC C_j is a strictly stoichiometric BC we solve the feasibility problem in Eq. (18), where $\mathbf{e}_j$ is the vector with a unit value for the jth entry and zero values elsewhere and **Y** being the stoichiometric map. The linear program will be feasible if $\mathbf{e}_j$ is a linear combination of rows in **Y**.

$$\begin{aligned} \underset{\mathbf{x}}{\text{minimize}} \quad & 0 \\ \text{subject to} \quad & \mathbf{Y}^{\mathrm{T}}\,\mathbf{x} = \mathbf{e}_j \end{aligned} \qquad (18) \quad .$$

Moreover, Küken et al. identified BCs under different sets of constraints, including the scenario where reaction reversibility is considered and canonical flux bounds are imposed and the scenario were the lower bound of the biomass reaction is set to at least 99% of its optimum obtained from flux balance analysis [15]. Comparing the sets of BCs found under the respective scenarios allows to identify type-II nonstoichiometric BCs, which are those complexes being balanced under imposed lower bound on biomass, but not under canonical flux bounds. The remaining BCs are then classified as stoichiometric BCs.

Supplementary Table

Table S1 Overview of network properties. Number of model species, reactions and complexes as well as percentage of balanced complexes identified in twelve genome-scale metabolic networks are presented. Blocked reactions were removed from the metabolic networks and do not count to the presented number of complexes.

Metabolic model	Number of model species	Number of reactions	Number of complexes	% balanced complexes	Computation time to identify balanced complexes [min]
A. niger **iMA871**	879	2402	1955	23.2	3.5
A. thaliana **AraCore**	407	1098	858	3.8	0.6
C. reinhardtii **iCre1355**	1220	3482	3147	15.4	11.4
E. coli **iJO1366**	1136	3364	2974	11.4	9.5
M. acetivorans **iMB745**	478	1156	1076	43.3	1
M. barkeri **iAF692**	454	1074	979	58.0	0.8
M. musculus	599	1720	1449	3.6	1.8
M. tuberculosis **iNJ661m**	592	1500	1379	28.6	1.5
N. pharaonis	465	1168	1044	1.8	1.1
P. putida **iJN746**	539	1304	1182	3.2	1.2
T. maritima **iLJ478**	328	760	708	40.7	0.5
S. cerevisiae **Yeast8**	1764	5556	4701	10.5	25.2

S3 Balancing calculations for the example in Fig. 1b of the main text

Claim

The network in Fig. 1b is going to have all complexes balanced, if $k_1 k_2 = k_3{}^2$.

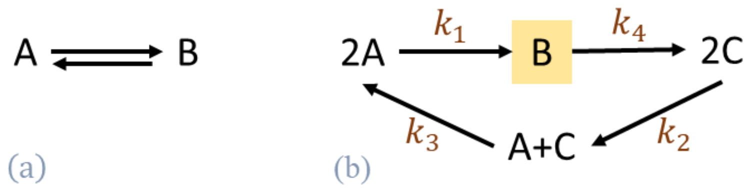

To demonstrate this, let us first write down the steady state equation for species A.

$$2k_1 x_A{}^2 - k_3 x_A x_C - k_2 x_C{}^2 = 0 \, .$$

Hence,

$$2k_1 \left(\frac{x_A}{x_C}\right)^2 - k_3 \frac{x_A}{x_C} - k_2 = 0 \, .$$

It follows that

$$x_A = \frac{k_3 + \sqrt{k_3{}^2 + 8k_1 k_2}}{4k_1} x_C \, .$$

On the other hand, from the steady state equation for B, one obtains

$$x_B = \frac{k_1}{k4} x_A{}^2 \, .$$

Therefore, x_C is the only degree of freedom in the steady state concentration vector **x**.

Next, let us assume $k_1 k_2 = k_3{}^2$; from the equation for x_A we obtain

$$x_A = \frac{k_3}{k_1} x_C = \frac{k_2}{k_3} x_C = \sqrt{\frac{k_2}{k_1}}\, x_C \, .$$

Now, we check the balancing condition for complexes:

(2A): $k_1 x_A{}^2 - k_3 x_A x_C = k_1 x_A{}^2 - k_3 x_A \left(\frac{k_1}{k_3} x_A\right) = 0$.

(A+C): $k_3 x_A x_C - k_2 x_C{}^2 = k_3 \left(\frac{k_2}{k_3} x_C\right) x_C - k_2 x_C{}^2 = 0$.

(2C): $k_2 x_C{}^2 - k_4 x_B = k_2 x_C{}^2 - k_1 x_A{}^2 = k_2 x_C{}^2 - k_1 (\sqrt{\frac{k_2}{k_1}}\, x_C)^2 = 0$.

Therefore, all complexes will have to be balanced at all steady states, under this particular condition on the rate constants. ■

Availability of code, data and material

The codes, as well as all networks used in the analysis are available here.

References

[1] S. Boyd and L. Vandenberghe, "Duality," in *Convex Optimization,* Cambridge University Press, 2004, pp. 215-288.

[2] A. Küken, P. Wendering, D. Langary and Z. Nikoloski, "A structural property for reduction of biochemical networks," *Scientific Reports,* vol. 11, no. 17415, 31 August 2021.

[3] M. R. Andersen, M. L. Nielsen and J. Nielsen, "Metabolic model integration of the bibliome, genome, metabolome and reactome of Aspergillus niger," *Molecular Systems Biology,* vol. 4, p. 178, 1 2008.

[4] A. Arnold and Z. Nikoloski, "Bottom-up Metabolic Reconstruction of Arabidopsis and Its Application to Determining the Metabolic Costs of Enzyme Production," *Plant Physiology,* vol. 165, pp. 1380-1391, 5 2014.

[5] S. Imam, S. Schäuble, J. Valenzuela, A. López García de Lomana, W. Carter, N. D. Price and N. S. Baliga, "A refined genome-scale reconstruction of Chlamydomonas metabolism provides a platform for systems-level analyses," *The Plant Journal,* vol. 84, pp. 1239-1256, 11 2015.

[6] J. D. Orth, T. M. Conrad, J. Na, J. A. Lerman, H. Nam, A. M. Feist and B. Ø. Palsson, "A comprehensive genome-scale reconstruction of Escherichia coli metabolism—2011," *Molecular Systems Biology,* vol. 7, p. 535, 1 2011.

[7] M. N. Benedict, M. C. Gonnerman, W. W. Metcalf and N. D. Price, "Genome-Scale Metabolic Reconstruction and Hypothesis Testing in the Methanogenic Archaeon Methanosarcina acetivorans C2A," *Journal of Bacteriology,* vol. 194, p. 855–865, 12 2012.

[8] A. M. Feist, J. C. M. Scholten, B. Ø. Palsson, F. J. Brockman and T. Ideker, "Modeling methanogenesis with a genome-scale metabolic reconstruction of Methanosarcina barkeri," *Molecular Systems Biology,* vol. 2, p. 2006.0004, 1 2006.

[9] L.-E. Quek and L. K. Nielsen, "ON THE RECONSTRUCTION OF THE MUS MUSCULUS GENOME-SCALE METABOLIC NETWORK MODEL," in *Genome Informatics 2008*, IMPERIAL COLLEGE PRESS, 2008, pp. 89-100.

[10] X. Fang, A. Wallqvist and J. Reifman, "Development and analysis of an in vivo-compatible metabolic network of Mycobacterium tuberculosis," *BMC Systems Biology,* vol. 4, p. 160, 2010.

[11] O. Gonzalez, T. Oberwinkler, L. Mansueto, F. Pfeiffer, E. Mendoza, R. Zimmer and D. Oesterhelt, "Characterization of Growth and Metabolism of the Haloalkaliphile Natronomonas pharaonis," *PLOS Computational Biology,* vol. 6, pp. 1-10, 6 2010.

[12] J. Nogales, B. Ø. Palsson and I. Thiele, "A genome-scale metabolic reconstruction of Pseudomonas putida KT2440: i JN746 as a cell factory," *BMC Systems Biology,* vol. 2, p. 79, 2008.

[13] Y. Zhang, I. Thiele, D. Weekes, Z. Li, L. Jaroszewski, K. Ginalski, A. M. Deacon, J. Wooley, S. A. Lesley, I. A. Wilson, B. Palsson, A. Osterman and A. Godzik, "Three-Dimensional Structural View of the Central Metabolic Network of Thermotoga maritima," *Science*, vol. 325, p. 1544–1549, 9 2009.

[14] H. Lu, F. Li, B. J. Sánchez, Z. Zhu, G. Li, I. Domenzain, S. Marcišauskas, P. M. Anton, D. Lappa, C. Lieven, M. E. Beber, N. Sonnenschein, E. J. Kerkhoven and J. Nielsen, "A consensus S. cerevisiae metabolic model Yeast8 and its ecosystem for comprehensively probing cellular metabolism," *Nature Communications*, vol. 10, p. 3586, 8 2019.

[15] J. D. Orth, I. Thiele and B. Ø. Palsson, "What is flux balance analysis?," *Nature biotechnology*, vol. 28, p. 245–248, 3 2010.
